# Boolean-network simplification and rule fitting to unravel chemotherapy resistance in non-small cell lung cancer

**DOI:** 10.1371/journal.pone.0357764

**Published:** 2026-09-15

**Authors:** Alonso Espinoza, Eric Goles, Marco Montalva-Medel

**Affiliations:** 1 Facultad de Ingenieria y Ciencias, Univesidad Adolfo Ibañez, Santiago, Chile; 2 Laboratoire d’Informatique et Systèmes, Centre National de la Recherche Scientifique, Aix-Marseille Univesité, Marseille, France; 3 Millennium Nucleus for Social Data Science (SODAS), Santiago, Chile; Texas A&M University, UNITED STATES OF AMERICA

## Abstract

Boolean networks are powerful frameworks for capturing the logic of gene-regulatory circuits, yet their combinatorial explosion hampers exhaustive analyses. Here, we present a systematic reduction of a published 31-node Boolean model that describes cisplatin- and pemetrexed-resistance in non-small-cell lung cancer to a compact 9-node core that exactly reproduces the original attractor landscape. Through a sequence of biologically guided reductions (31→29→14→9 nodes), the streamlined network shrinks the state space by four orders of magnitude, enabling rapid exploration of critical control points, rules fitting, and candidate therapeutic targets. Extensive synchronous and asynchronous simulations, combined with a Boolean rule-fitting algorithm that removes spurious limit cycles, confirm that the three clinically relevant steady states and their basins of attraction are conserved and reflect resistance frequencies close to those reported in clinical studies. The reduced model provides an accessible scaffold for future mechanistic and drug-discovery studies.

## Introduction

Non-small cell lung cancer (NSCLC) is a type of cancer that accounts for approximately 85% of all lung cancer cases [1]. It is a heterogeneous disease that includes several subtypes such as adenocarcinoma, squamous cell carcinoma, and large cell carcinoma. The primary risk factor for developing NSCLC is smoking, although other factors such as radon exposure and air pollution have also been identified [1]. Most patients are diagnosed in advanced stages due to the lack of effective screening programs and the late onset of clinical symptoms, resulting in a generally unfavorable prognosis.

This study focuses on the dynamical analysis of a Boolean genetic network designed to investigate drug resistance mechanisms in NSCLC. This network, originally proposed in a recent study, includes interactions between long non-coding RNAs (lncRNAs), microRNAs (miRNAs), and various transcription factors that collectively influence resistance to chemotherapeutic treatments such as cisplatin and pemetrexed [2]. The structure and dynamics of the network were modeled using a Boolean network approach, which allows the simulation of genetic regulation through binary states (active/inactive), thus facilitating the study of network stability and response under different update schemes.

Boolean networks (BNs) are widely recognized for their ability to capture gene interactions and the overall dynamic behavior of genetic regulatory networks, making them a popular model for inferring regulatory structure from gene expression data. These approaches are particularly useful in situations where gene expression measurements are noisy and can lead to inconsistent observations [3].

Boolean genetic networks, which simplify the complexity of biological interactions into binary relationships, emerge as powerful tools to decode the multifaceted dynamics of drug resistance. By modeling these interactions within the context of NSCLC, this study aims not only to identify key network configurations that promote resistance but also to evaluate how modifications in these configurations can alter cellular phenotypes. This approach can reveal novel therapeutic targets and offer insight into the overcoming of chemotherapy resistance.

Analyzing all deterministic dynamics of a Boolean regulatory network is a challenging problem, as it grows exponentially with the number of nodes. In this context, mathematical and computational tools, such as equivalence classes of update schemes, are introduced to study the dynamic robustness under different update schemes [4,5]. These tools are essential for discovering new information related to the model’s attractors, considering all possible deterministic dynamics (i.e., not only the synchronous dynamics that is typically used in these models).

Moreover, studies such as the one conducted by Aracena et al. [6] have demonstrated the efficacy of update scheme tools in revealing the complex dynamics of BNs. Although these tools are not specifically designed to evaluate stability in the traditional sense of stability analysis, they offer valuable resources for investigating how the sequence of updates influences the overall stability of systems modeled as BNs. This is particularly useful in systems whose dynamics are sensitive to the order in which their components are updated.

From a biological standpoint, the central question motivating this work can be stated as follows: can cisplatin/pemetrexed resistance in NSCLC be captured by a minimal regulatory core that preserves the clinically relevant phenotypes (drug resistance, senescence, and apoptosis), and what does this minimal core imply for the identification of candidate biomarkers and therapeutic targets? To address it, we combine the theoretical tools introduced above with the biologically grounded Boolean model of NSCLC drug resistance proposed by Gupta et al. [2], whose work focused on the construction and synchronous analysis of the full 31-node network. Specifically, we (i) reconstruct in logical form the 31-node Boolean network of [2] and validate its reported steady states under synchronous update; (ii) perform a sequence of biologically motivated reductions (31→29→14→9 nodes) that preserve the attractor landscape and yield a minimal core network; (iii) use update-digraph theory to exhaustively characterize the distinct deterministic update schemes of the minimal 9-node model; and (iv) introduce a Boolean rule-fitting algorithm that removes spurious attractors while retaining the clinically relevant phenotypes, increasing the robustness of the model across update schemes. Together, these steps provide a compact yet dynamically faithful representation of chemotherapy resistance mechanisms in NSCLC.

## Materials and methods

### Data acquisition

The Boolean network used in this study originates from the scientific article “A dynamic Boolean network reveals that the BMI1 and MALAT1 axis is associated with drug resistance by limiting miR-145-5p in non-small cell lung cancer” [2].

### Data analysis

The R programming language (version 4.3.3) [7] was used for synchronous Boolean-network simulations, primarily through the BoolNet package [8]. The synchronous simulation workflow was implemented to read Boolean rules in BoolNet-compatible format, compute attractors and attraction-basin sizes, and evaluate the relative basin contribution of each phenotype. The same workflow was also used for *in silico* perturbation assays by forcing selected nodes to remain active or inactive during the simulations. These analyses were applied to the reconstructed and reduced NSCLC BNs, including the reconstructed network without terminal phenotype outputs, the intermediate reduced networks, the minimal 9-node core, and the final rule-fitted 9-node network.

Python programming language (version 3.10.5) [9] was used for complementary computational analyses, visualization, deterministic asynchronous-update simulations, and Boolean rule fitting. The Python workflow included routines for drawing regulatory graphs, constructing attraction-basin maps for the 9-node networks, enumerating representative deterministic update schemes through update-digraph equivalence classes, and simulating attractors and basins for each representative update scheme. Python was also used to perform exhaustive Boolean rule fitting in the 9-node core network, identifying candidate rules that preserve the biologically relevant steady states while eliminating undesired synchronous attractors. The main Python libraries employed were NumPy, pandas, NetworkX, matplotlib, pyvis, pydot, and Graphviz-related packages.

For synchronous attraction-basin analyses, exhaustive enumeration of the $2^{n}$ initial configurations was performed for the reduced networks for which this procedure was computationally feasible, including the 9- and 14-node networks. The 9-node attraction-basin maps were obtained by enumerating all 2^9^ = 512 states and assigning each initial condition to its corresponding attractor under the specified update scheme. For deterministic asynchronous dynamics, block-sequential update schedules were grouped into update-digraph equivalence classes, and one representative per class was simulated to quantify the robustness of the attractor landscape under distinct deterministic update schemes.

The Boolean rule-fitting procedure was applied to the minimal 9-node network. Candidate Boolean expressions were exhaustively evaluated to identify local update rules that preserved the desired fixed points associated with Drug Resistance, Senescence, and Apoptosis while removing spurious attractors. For each potential target node, candidate regulators were allowed to be drawn from the other eight network nodes, rather than only from the node’s existing parents in the original regulatory graph. Self-regulation was not permitted; therefore, the target node was excluded from its own candidate regulator set. All 9 nodes were tested as possible targets for rule fitting; the search was not restricted a priori to nodes suspected to participate in the spurious cycle. Candidate rules were generated from combinations of one to three regulators and were tested through full-network simulations. The selected fitted rule was then incorporated into the final 9-node model and re-evaluated under both synchronous and deterministic asynchronous update schemes. The same exhaustive rule-fitting strategy was not applied directly to the reconstructed 31-node network because its combinatorial search space would require additional pruning or heuristic optimization.

To facilitate reproducibility, the complete computational workflow was consolidated in a single public repository containing the Boolean rule definitions, simulation scripts, visualization routines, deterministic update-scheme analysis, rule-fitting implementation, and reproducibility instructions. The repository provides the model definitions in BoolNet-compatible plain-text format, together with the code required to reproduce the synchronous simulations, network visualizations, attraction-basin analyses, deterministic asynchronous update-scheme results, and fitted-rule validation. The reproducibility repository corresponding to the revised manuscript is available at https://github.com/aer-neo/Boolean-Network-NSCLC-Pipeline/tree/bd5cbb6. The associated commit hash is bd5cbb6.

As a practical reference for reproducibility, the deterministic asynchronous update-scheme analysis required approximately 20 minutes in Google Colab for the original 9-node network and approximately 25 minutes for the rule-fitted 9-node network. These runtimes correspond to the enumeration of representative update schemes through update-digraph equivalence classes and the subsequent attractor/basin simulation for each representative scheme. Actual execution times may vary depending on the computational resources allocated by Google Colab at runtime.

### Boolean networks

The BNs are simple yet powerful models introduced decades ago [10] to represent complex systems. In these models, biological components are represented by binary states (“0” or “1”), which helps to simplify dynamic processes and offer insights into their behavior. BNs have been used extensively to study Gene Regulatory Networks (GRNs) across various species (e.g., [5,11–14]). A formal BN consists of a finite directed graph where nodes correspond to biological components, each having a discrete state of either 0 (inactive) or 1 (active), and the edges represent the interactions between these components. The states of the nodes are updated according to Boolean functions, following a predefined update schedule. For a network with *n* nodes, we represent an update schedule by a function s:{1,...,n}→{1,...,n}, where *s*(*i*) < *s*(*j*) indicates that node *i* is updated before node *j*. These update schemes have proven to be useful for analyzing various biological applications (e.g., [5,11–14]) as well as for developing new theoretical results in discrete mathematics (e.g., [15–17]). In this work, we will use the following ones:

- (Deterministic) Synchronous update schedule or parallel update: *s*(*k*) = 1 for all nodes *k* at each time step; that is, all nodes are updated simultaneously. We denote this by *s* = (1,2,...,*n*), meaning that all nodes belong to a single update block.
- (Deterministic) Asynchronous update schedule or block sequential update schedule: if *A*_1_,...,$A_{k}$ is a partition of the set of nodes {1,...,*n*}, 1<k≤n, then *s*(*j*) = *i*, for all $j\in A_{i}$, at each time step. In other words, at each time step, the blocks of nodes $A_{i}$ are updated one by one (sequentially), and the nodes inside each block are updated in parallel. Notation: $s=(A_{1})(A_{2})\cdots (A_{k})$.

The above implies that a specific dynamic behavior of the network is determined by the choice of an update schedule. This is typically represented by a state transition graph, where each node corresponds to a configuration (or vector of states) of the network’s binary states, and the arcs represent transitions between configurations at each time step, resulting from the application of local Boolean functions according to the update schedule considered. Additionally, since there are $2^{n}$ possible configurations, the network will always evolve toward a subset of configurations known as attractors. These attractors can be either *steady states* (also called *fixed points*), where the configuration remains unchanged after applying the Boolean functions, or *limit cycles* with a period *l* > 1 that is, repeating sequences of *l* configurations $\{x(0),x(1),...,x(l-1)\}\subseteq \{0,1{\}}^{n}$, where each *x*(*t*) transitions to x(t+1(modl)). Intuitive*l*y, a *l*imit cycle of length *l* means the ne*t*work oscillates periodically through *l* distinct states without ever sett*l*ing on a single one. The *attraction basin* (or *basin of attraction*) of an attractor is the set of all $2^{n}$ configurations that eventua*ll*y converge to that attractor under the chosen update schedule. Throughout this manuscript, basin sizes are reported as percentages of the total $2^{n}$ configurations (obtained by exhaustive enumeration for n≤29 or by uniform random sampling otherwise). For brevity, we hereafter use “steady state” as the preferred term for fixed-point attractors.

In this context, a 31-node Boolean Network model for the study of drug resistance mechanisms in NSCLC was introduced in [2], where the authors employed a synchronous update scheme and that we reconstruct in this manuscript with some small adjustments (see S1 Fig, the corresponding local Boolean functions in S1 Table in S1 File, and the reconstruction details in Network Reconstruction) and then reduce it (see Importance of Complexity Reduction in the Discussion) while considering all the deterministic update schedules explained above. This represents a large family of schemes that grows exponentially as the network size increases [15]. Specifically, the number $T_{n}$ of deterministic update schedules corresponding to a directed graph with *n* vertices is given by:


Tn=∑k=0n−1(nk)Tk,

where $T_{0}\equiv 1$. Additionally, if we consider that the dynamics obtained by each of these $T_{n}$ schemes generates $2^{n}$ possible configurations, the computational effort required for an exhaustive analysis of all these dynamics (which we conduct in this study) becomes even more demanding. In practice, such an analysis can only be performed for small values of *n*; therefore, in Update digraphs and different dynamics in BNs, we outline the key concepts of update digraph theory, which significantly reduce these computational costs.

### Network reduction pipeline

The four-stage reduction 31→29→14→9 nodes used in this study follows a sequence of well-defined criteria that combine algorithmic detection with manual biological curation. The pipeline can be summarized as the following ordered steps:

1. **Detection of dynamically redundant nodes** (31→29). For each node, we check whether removing it (and propagating its expression into the rules of its targets) preserves both the set of attractors and their basin sizes under synchronous update. Nodes that pass this test *and* whose biological role is already covered by parallel pathways in the network are flagged as redundant. This step is partly algorithmic (single-node deletion test) and partly manual (biological assessment of pathway redundancy).
2. **Fixing the chemotherapeutic context.** The input node DNA_Damage is fixed at value 1, modeling the persistent DNA damage induced by cisplatin/pemetrexed exposure. This is a *contextual* choice that determines the scope of the subsequent reductions.
3. **Removal of nodes with fixed configurations** (29→14). Under DNA_Damage = 1, all nodes whose state becomes constant across every attractor are detected and removed; their fixed value is hard-coded into the rules of their downstream targets. This step is fully algorithmic.
4. **Removal of terminal node groups** (14→9). Nodes (or groups of nodes) whose state is fully determined by upstream regulators and that do not feed back into the regulatory core are identified as “terminal” and pruned. The fully algorithmic part is the detection of zero out-degree (within the strongly connected core); the choice of which terminal groups to aggregate vs. remove is manual.

After each step we verify that (a) the set of steady states associated with the clinically relevant phenotypes (drug resistance, senescence, apoptosis) is preserved, and (b) basin sizes vary only within the limits expected from the dimensionality reduction. Steps 1 and 4 require biological judgment, while steps 2 and 3 are fully algorithmic given the chosen context. We comment on the generalizability of this pipeline to other BNs in Scalability.

#### Scope of the reduced models.

Because steps 2–4 are carried out under the assumption DNA_Damage = 1, the resulting 14- and 9-node networks are intended for scenarios with *persistent* drug-induced DNA damage. They are *not* suitable for studying damage initiation, DNA repair, or drug-withdrawal dynamics. The 31- and 29-node versions retain the full DNA_Damage axis and remain available for those broader contexts.

### Update digraphs and different dynamics in BNs

#### Intuition.

The number of deterministic update schedules grows exponentially with the network size, but many of them yield exactly the same dynamics. Update digraphs provide a compact way to encode the *relative timing* between interacting nodes: for each regulatory arc, they record whether the source node is updated before or after its target. The key consequence is that schedules sharing the same update digraph produce identical state-transition dynamics. Thus, instead of simulating every possible schedule separately, we simulate one representative per update-digraph equivalence class, reducing the computational burden while still covering all distinct deterministic dynamics. The formal definitions are given below; the detailed toy example, including the full transition tables, is provided in Supporting information.

Here we provide a summary of the key concepts and results from [4,6,18,19], which facilitate the classification of deterministic update schemes that produce identical dynamics. Let *G* = (*V*, *E*) be a directed graph, *s* an update schedule, and the labeling function $lab_{s}:E\to \{-,+\}$ defined as:


$$\begin{array}{c} \forall (i,j)\in E,\;lab_{s}(i,j)=\left\{ \begin{array}{cc} + & \text{if }s(i)\geq s(j),\\ - & \text{if }s(i)<s(j). \end{array}\right. \end{array}$$

By assigning the labels + or – to each arc (*i*,*j*) in *E*, we obtain a labeled directed graph (*G*, *lab*). When $lab=lab_{s}$ for some update schedule *s*, the labeled graph $\left(G,lab_{s}\right)$ is referred to as the update digraph. It is important to note that while every update digraph is a labeled graph, the reverse is not necessarily true. In [4], the following characterization of update digraphs was established:

**Theorem 1.** *A labeled digraph is an update digraph if and only if reversing the direction of the arcs labeled – results in a new digraph (possibly a multigraph) that contains no cycles with – labeled arcs.*

Additionally, in [18] the following result was proven:

**Theorem 2.** *Let* $N_{1}=(G,F,s_{1})$ *and* $N_{2}=(G,F,s_{2})$ *be two BNs (i.e., they share the same graph G and global function F, but differ only in their update schemes s_1_ and s_2_). If their update digraphs are identical, i.e.,* $\left(G,lab_{s_{1}})=(G,lab_{s_{2}}\right)$*, then N*_*1*_ *and N*_*2*_ *exhibit the same dynamics.*

Using Theorem 2, we can define equivalence classes of update schemes that lead to the same update digraph, and therefore produce identical dynamics:


$$\left[s{]}_{G}=\{s^{\prime }:\;(G,lab_{s})=(G,lab_{s^{\prime }})\right\}$$

This formulation establishes a bijection between update digraphs and these equivalence classes, which partition the set of all $T_{n}$ update schemes. Consequently, this allows the study of distinct network dynamics by focusing only on a subset of update schemes, specifically those that serve as representatives of each equivalence class. An algorithm for determining these representative schemes for each class was introduced in [6].

The following small example illustrates the reduction obtained by this equivalence relation without placing the full enumeration in the main text.

**Example 1.** *Consider the digraph G = (V,E) where V = {A,B,C} and E = {(A,C),(C,B),(B,C),(C,A)}.*

For this graph, the *T*_3_ = 13 deterministic update schedules collapse into only 9 update-digraph equivalence classes. Therefore, to exhaustively study the distinct deterministic dynamics of any Boolean network with this interaction graph, it is sufficient to simulate 9 representative schedules rather than all 13 schedules. The complete equivalence classes and their associated update digraphs are reported in S2 Table in S1 File and S2 Fig, respectively; the corresponding transition tables and state-transition graphs for one concrete Boolean network on this graph are shown in S3 Table in S1 File and S3 Fig. This example illustrates the practical role of update digraphs in the present study: they allow us to test robustness across all distinct deterministic update dynamics while avoiding redundant simulations.

In the theory of update digraphs presented in [4,6,18,19], additional properties are established that explain why the reduction in the number of schemes required to analyze the distinct dynamics of a Boolean network is typically much more significant than the 31% reduction observed in this example, particularly as the network size increases.

In this way, we will explore what happens beyond the parallel scheme, which may be biased [20] or vulnerable to perturbations in the schemes, as is the case with certain families of BNs, such as elementary cellular automata. In these networks, some can exhibit strong robustness while others may not [16,17,21,22].

## Results

### Network reconstruction

The gene regulatory network used in this study was reconstructed from the work of Gupta et al. [2]. Originally designed to investigate mechanisms of drug resistance in NSCLC, this network includes 31 nodes representing key biological components such as long non-coding RNAs, microRNAs, and transcription factors. These interact to influence resistance to chemotherapies such as cisplatin and pemetrexed. The interactions between nodes were modeled using Boolean functions detailed in S1 Table in S1 File of the original article, while the complete network is illustrated in S1 Fig.

Following reconstruction, initial simulations under a synchronous update scheme revealed the presence of four terminal phenotypes or stable states: proliferation, drug resistance, senescence, and apoptosis, along with three limit cycles. These cycles correspond to periodic oscillations in node configurations and are described in S4 Table in S1 File. The original article from which the network was derived does not indicate whether these limit cycles fulfill a biological function; therefore, they are assumed to be spurious cycles. The initial analysis validated that the reconstructed Boolean functions preserve the original state configurations, confirming the accuracy of the reconstructed network.

### Reduction to a 29-node network

The main objective was to simplify the network while maintaining the essential dynamics that determine the key phenotypes. Following the reduction pipeline described in Network reduction pipeline, we first evaluated whether any individual node could be removed from the 31-node network without altering the relevant synchronous attractor landscape. For each candidate node *v*, we removed *v*, propagated its Boolean expression into the update rules of its targets, and recomputed the synchronous dynamics of the resulting reduced network. This single-node deletion analysis showed that no individual deletion was sufficient to preserve the desired dynamical behavior while removing the spurious oscillatory dynamics.

We therefore proceeded to a second, systematic pairwise-deletion step focused on the nodes involved in the length-4 limit cycles reported in S4 Table in S1 File. Specifically, we selected as candidates those nodes whose values were not constant across these cycles, namely HDAC1, Myc, Mdm2, p53_K, Sirt_1, p53_INP1, Wip1, p21, CDK46_CycD, CDK2_CycE, and RB. The rationale was to identify node pairs whose removal would eliminate the length-4 cycles and redirect the corresponding configurations toward biologically relevant steady-state attractors, while preserving the configurations of the remaining nodes in those attractors.

Among the tested pairs, the removal of Sirt_1 and p53_INP1 satisfied this criterion. In the resulting 29-node network, the four terminal phenotypes were preserved, the state configurations of the remaining regulatory nodes in the steady states were unchanged, and the spurious length-4 cycles were eliminated. Because DNA_Damage was inactive in all states of these length-4 cycles, the corresponding configurations were associated with the no-damage/proliferative context and, after the removal of Sirt_1 and p53_INP1, converged to the proliferation steady-state attractor. Thus, in this work the term “redundant” refers to a joint dynamical redundancy: Sirt_1 and p53_INP1 were not identified as individually dispensable nodes, but as a pair whose regulatory contribution was not required to preserve the relevant steady-state phenotypes and whose removal simplified the oscillatory landscape. Biological interpretation was then used to support this dynamical result, since both nodes act through regulatory effects on the p53-related module that are also represented by parallel routes in the network.

**Fig 1:**
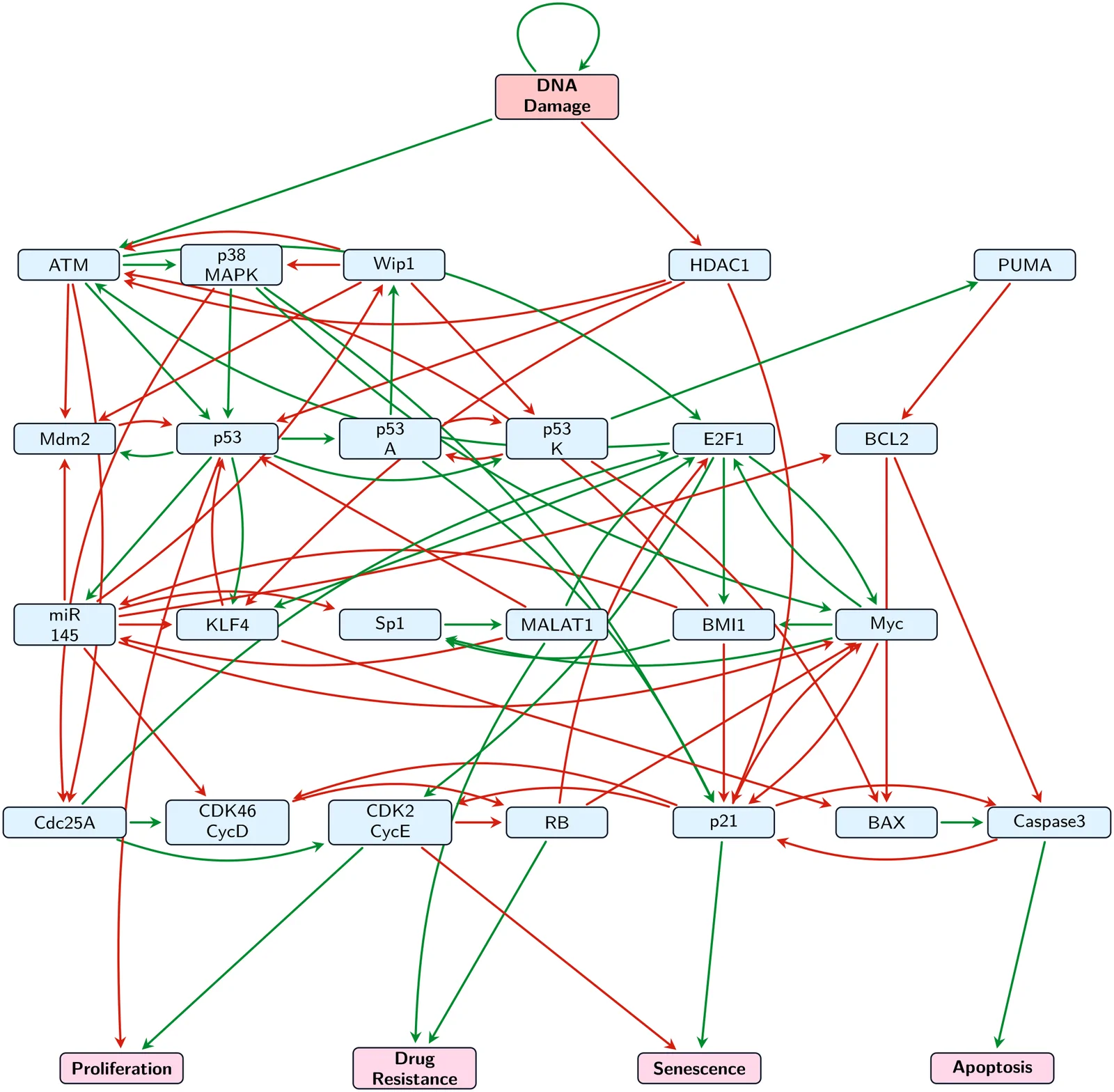
Reduced Boolean network for NSCLC. For the construction of this network, the 31-node network was simplified with the most relevant genes and built based on the information in S5 Table in S1 File, resulting in a total of 29 nodes. Green arcs represent positive weights (activations), and red arcs represent negative weights (inhibitions). The red-colored node represents “DNA_Damage”, which corresponds to the input node; the four phenotypes that can be generated in this network are shown in pink, and the biological components corresponding to long non-coding RNAs, microRNAs, and various transcription factors are in blue.

The reduced 29-node Boolean network maintained the four terminal phenotypes while simplifying its dynamical behavior by reducing the number of limit cycles to a single cycle of length 2. This network exhibited a total of 2^25^ = 33,554,432 possible configurations, from which 50% of the initial states converged to steady state 1 (proliferation), 49.89% to steady state 2 (drug_resistance), 0.01% to steady state 3 (senescence), 0.04% to steady state 4 (apoptosis), and 0.05% to limit cycle 1. Importantly, this reduction preserved the behavior of key regulatory nodes, ensuring that the essential functional characteristics of the system remained intact. Furthermore, the attractor landscape of the reduced network was consistent with that of the original network, demonstrating that the reduction process did not alter the fundamental properties of state convergence. The results of this analysis are summarized in Table 1, while the structure of the reduced network is depicted in Fig 1. For the simulation of this 29-node network, the rules of the 31-node network were adapted, and these new rules are shown in S5 Table in S1 File.

**Table 1:** Attractors results for the 29-node network simulation.

Components	Steady state 1	Steady state 2	Steady state 3	Steady state 4	Limit Cycle 1
ATM	0	1	1	1	1 1
p38MAPK	0	1	1	1	1 1
miR_145	0	0	1	1	1 1
Sp1	1	1	0	0	0 0
MALAT1	1	1	0	0	0 0
BMI1	1	1	0	0	0 0
KLF4	1	1	0	0	0 0
HDAC1	0	0	0	0	0 0
Myc	1	0	0	0	0 0
p53	0	0	1	1	1 1
Mdm2	1	0	0	0	0 0
p53_A	0	0	0	1	1 0
p53_K	0	0	1	0	1 0
Wip1	0	0	0	0	0 0
p21	0	0	1	1	0 1
Cdc25A	1	0	0	0	0 0
CDK46_CycD	1	0	0	0	0 0
CDK2_CycE	1	0	0	0	0 0
RB	0	1	1	1	1 1
PUMA	0	0	1	0	0 1
BCL2	1	1	0	0	0 0
BAX	0	0	0	1	1 0
E2F1	1	1	0	0	0 0
Caspase3	0	0	0	1	0 1
DNA_Damage	0	1	1	1	1 1
**Proliferation**	1	0	0	0	- -
**Drug Resistance**	0	1	0	0	- -
**Senescence**	0	0	1	1	- -
**Apoptosis**	0	0	0	1	- -
**Attraction basin**	50.00	49.89	0.01	0.04	0.05

### Reduction to a 14-node network

In a second simplification stage, additional nodes were reduced whose configuration was fixed or deducible from other nodes in the context of activated “DNA_Damage” (value 1). We emphasize that this and the subsequent reduction are derived under the persistent-DNA-damage assumption (DNA_Damage = 1); the resulting 14- and 9-node networks therefore describe regulatory dynamics during sustained drug exposure and are *not* intended to model damage initiation, DNA repair, or drug withdrawal. The resulting 14-node network, described in S4 Fig, continued to preserve the dynamics observed in the 31- and 29-node networks. In this case, the simulation led to three stable states and one limit cycle as shown in S6 Table in S1 File. The size of the configuration space was drastically reduced, facilitating a more efficient computational analysis.

For the simulation of this 14-node network, the rules of the 29-node network were adapted, and these new rules are shown in S6 Table in S1 File. This led to a total of 2^14^ = 16,384 possible configurations, with 98.44% converging to steady state 1 (drug_resistance), 0.39% to steady state 2 (senescence), 0.39% to steady state 3 (apoptosis), and 0.78% to the limit cycle. These values are shown in S7 Table in S1 File. Upon analyzing the configurations of the nodes obtained and comparing them with the 31-node and 29-node networks, no changes in their values were observed, indicating that the dynamics remain unaffected.

### Reduction to a 9-node network

**Fig 2:**
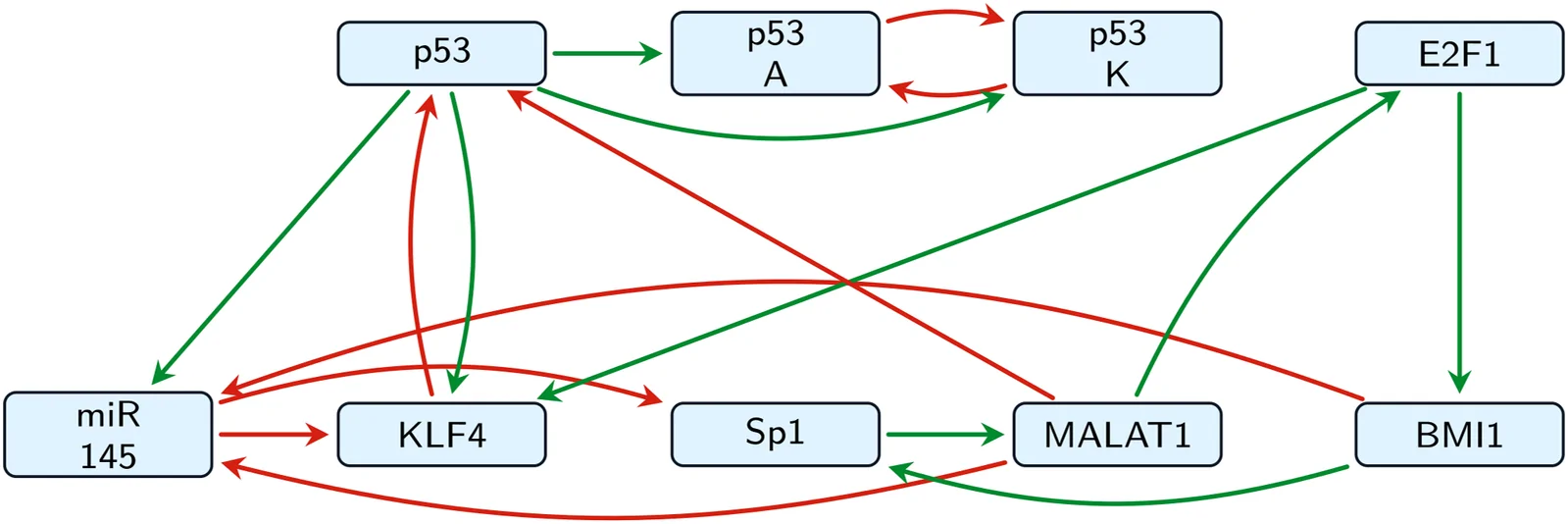
Minimal 9-node Boolean network for NSCLC. To construct this network, the 14-node network was simplified from the information in S8 Table in S1 File, discarding nodes that can be deduced from their configurations by other nodes. Green arcs represent positive weights (activations), and red arcs represent negative weights (inhibitions).

Finally, a minimally complex network comprising only 9 nodes was obtained, as shown in Fig 2. For this final reduction, all nodes exhibiting terminal‐node group behavior were removed, that is, groups of terminal nodes whose states are fully determined by upstream components and do not feed back into the regulatory core. In S4 Fig, when identifying the nodes “p53_K,” “KLF4,” “miR_145,” “p53_A,” and “BMI1,” these point to the group “BAX,” “Caspase3,” “p21,” “BCL2,” and “PUMA,” the latter exhibiting terminal‐node group behavior. These nodes were eliminated, and the rules were modified as presented in S8 Table in S1 File.

In the synchronous simulation, this yielded a total of 2^9^ = 512 possible configurations, of which 98.44% converged to steady state 1 (drug resistance), 0.39% to steady state 2 (senescence), 0.39% to steady state 3 (apoptosis), and 0.78% to the length two limit cycle. When comparing these outcomes with those from the 31, 29, and 14 node networks, no differences were observed, as shown in Table 2. The steady state associated with the “Drug_Resistance” phenotype exhibited a significantly larger attractor basin relative to the others; these basins of attraction are illustrated in S5 Fig

**Table 2:** Attractors results for the 9-node network simulation.

Components	Steady state 1	Steady state 2	Steady state 3	Limit Cycle 1
miR_145	0	1	1	1 1
Sp1	1	0	0	0 0
MALAT1	1	0	0	0 0
BMI1	1	0	0	0 0
KLF4	1	0	0	0 0
p53	0	1	1	1 1
p53_A	0	0	1	0 1
p53_K	0	1	0	0 1
E2F1	1	0	0	0 0
**Attraction basin**	98.44	0.39	0.39	0.78

### Analysis of update schemes

To evaluate the robustness of the reduced 9-node network, the method described in Update digraphs and different dynamics in BNs was applied, identifying 10,632 representative deterministic update schemes. These representatives cover *all* equivalence classes under the update-digraph relation (Theorem 2), so that every distinct deterministic dynamic of the network is captured by exactly one class. Among these, 7,356 (69.19%) schemes exhibited only the three steady states, while the remaining 3,276 (30.81%) included limit cycles. Of the schemes with cycles, 2,836 exhibited a single limit cycle, 362 included two limit cycles, and 78 showed five limit cycles.

The analysis of these schemes, summarized in S9 Table in S1 File, revealed that the three steady states were preserved in all cases, demonstrating the robustness of the network under different update dynamics. Regarding the limit cycles, all corresponded to two-state oscillations (cycles of length 2), with the most frequent one matching the cycle observed in the synchronous simulation, as detailed in S10 Table in S1 File.

The representative update-scheme classes, raw attractor and basin-size outputs, and summary statistics used for the original 9-node network analysis are provided in S2–S4 Files, respectively.

### Rules fitting

In order to identify simpler or alternative rules that preserve the network’s expected dynamic behavior (that is, only the three steady states), we developed the Boolean Rule‐Fitting Algorithm. This optimized, exhaustive fitting method explores progressively larger combinations (ranging from one to three candidate regulatory nodes) one combination at a time, constructing Boolean expressions via the logical operators AND, OR, and NOT. Each candidate rule is evaluated in two stages: first, its local functionality is verified by checking whether it correctly reproduces the node’s value in the original attractors; then, if it passes this test, its global consistency is assessed by simulating the complete network dynamics under the new rule to confirm that the resulting attractors match exactly those desired. Further details on the combinatorial search space and computational implementation are provided in [23]; the pseudocode is provided in Supporting information.

For each of the 9 nodes of the minimal network, the algorithm explores candidate Boolean expressions whose regulators are drawn from the other eight nodes (i.e., not restricted to the target node’s current parents). Self-loops are not permitted, so the target node is excluded from its own candidate rule. All 9 nodes were tested as potential targets for rule fitting. The exhaustive search yielded 52 dynamically admissible rule substitutions across all target nodes. From this full computational output, five candidates were shortlisted because their regulatory logic was also biologically coherent with the established NSCLC network. These candidates correspond to biological-evidence ranks 1–5 and are reported in S11 Table in S1 File. The rank-1 BMI1 rule is listed first because it was carried forward into the fitted network, followed by the MALAT1 rule at rank 2, the miR-145 rule at rank 3, and the two p53 alternatives at ranks 4 and 5. Thus, the five rules denote the biologically prioritized subset used for detailed comparison, rather than the total number returned by the exhaustive search. The selected BMI1 rule is “(!p53_A&!p53_K)|E2F1” and is integrated into the fitted 9-node network shown in S12 Table in S1 File. Its selection combined *both* dynamical and biological criteria: it had to (i) produce exactly the desired attractor set under synchronous update and remove the spurious cycle, and (ii) preserve experimentally supported regulatory logic. The other four shortlisted rules remain plausible alternatives but were not substituted into the final fitted network.

In the synchronous simulation with the selected rule, this yielded a total of 2^9^ = 512 possible configurations, of which 99.22% converged to steady state 1 (drug resistance), 0.39% to steady state 2 (senescence), and 0.39% to steady state 3 (apoptosis). The results of this simulation are presented in S13 Table in S1 File.

As with the reduced 9-node network, the method described in Update digraphs and different dynamics in BNs was applied, identifying 23,107 representative deterministic update schemes that cover all equivalence classes of the rule-fitted network. The increase from 10,632–23,107 classes is a consequence of the modified BMI1 rule, which changes the set of edges in the regulatory graph: because update digraphs are defined over the graph’s arc set, altering the graph topology (i.e., the incoming edges of BMI1) changes the number of structurally distinct labelings and, consequently, the number of equivalence classes. Of these, 21,858 schemes (94.59%) exhibited only the three steady states, while the remaining 1,249 schemes (5.41%) included limit cycles. The analysis of these schemes, summarized in S14 Table in S1 File, revealed that the three steady states were preserved in all cases, demonstrating the robustness of the network under various update dynamics. Regarding the limit cycles, 586 schemes exhibited a single limit cycle, 534 included two limit cycles, and 129 showed three limit cycles. Furthermore, all detected cycles were of length 2, as detailed in S15 Table in S1 File. The representative update-scheme classes, raw attractor and basin-size outputs, and summary statistics used for the rule-fitted 9-node network analysis are provided in S5–S7 Files, respectively.

Importantly, the larger number of representative update schemes in the fitted network does not indicate reduced robustness. It reflects the finer update-digraph partition induced by the two additional regulatory arcs entering BMI1. Dynamically, the fitted network is more robust, since the fraction of update-digraph classes producing only steady states increases from 69.19% to 94.59%.

An empirically notable property is that *all* limit cycles observed in both the original and the rule-fitted 9-node networks are of length 2, both before and after rule fitting. One structural reason is the small diameter of the 9-node graph and the predominance of positive feedback loops in the regulatory core; long cycles require sufficient negative-feedback delay, and the minimal network lacks the chain length to sustain oscillations of period >2 under deterministic update. We note, however, that this is an empirical observation from exhaustive enumeration, not a formal impossibility theorem; longer cycles might in principle appear if the network were extended with additional negative feedback.

### Key biological insights

Before turning to the detailed Discussion, we summarize the main biological insights that emerge from the dynamical analysis of the reduced and fitted networks. Across all reduced networks (29-, 14-, and 9-node) and under both synchronous and asynchronous update regimes, the steady state associated with the “Drug Resistance” phenotype consistently exhibits the largest basin of attraction, reaching 98.44% in the 9-node network and 99.22% after rule fitting under synchronous update. This dominance qualitatively mirrors the high frequency of non-response to cisplatin/pemetrexed reported in the clinical literature [24]. At the same time, the reduction identifies a minimal regulatory core of nine nodes (miR_145, Sp1, MALAT1, BMI1, KLF4, p53, p53_A, p53_K, and E2F1) that is sufficient to recapitulate the three clinically relevant attractors of the original 31-node model (drug resistance, senescence, and apoptosis) under persistent DNA damage (DNA_Damage = 1).

Within this core, the E2F1–BMI1–p53 axis emerges as a dynamical bottleneck. The E2F1 branch of the fitted BMI1 rule, (!p53_A&!p53_K)|E2F1, is supported by direct promoter-level evidence for E2F1-mediated BMI1 activation [25], whereas the !p53_A&!p53_K branch is interpreted as BMI1 derepression when both phosphorylated p53 nodes are inactive. This p53-related branch is a modeling abstraction informed by evidence for a p53–miR-200c–BMI1 pathway in mammary epithelial and breast cancer systems [26], rather than a demonstrated direct interaction in NSCLC. Independent NSCLC experiments connect BMI1/miR-145-5p/Sp1 signaling with EMT and pemetrexed resistance [27]. Replacing the original BMI1 rule with this fitted rule abolishes the spurious limit cycle and increases the proportion of update schemes that yield only steady states from 69.19% to 94.59%.

From a therapeutic perspective, the compactness and robustness of the minimal core identify miR_145, MALAT1, BMI1, Sp1, and the p53 phosphorylation axis as candidate targets for shifting cellular fate from drug resistance toward senescence or apoptosis. These predictions are intended to generate experimentally testable hypotheses and require dedicated validation before any translational interpretation.

## Discussion

In Boolean network simulations, the configurations can reach steady states represented in this case as distinct phenotypes. Additionally, configurations can reach limit cycles, which are periodic oscillation patterns among a set of configurations. For example, a limit cycle of length 2 implies an alternation between two configurations. Configuration refers to the state of each node or biological component, indicating whether it is activated (1) or deactivated (0). For instance, each steady state or phenotype, as in Table 1, has its own node states. A network may have multiple configurations. The total number of configurations is calculated by raising the number of possible states for each node (in this case, two for states 0 and 1) to the total number of nodes in the network. For example, a network with 3 nodes would have 2^3^ = 8 possible configurations. These configurations can converge to a steady state or a limit cycle, which can be visualized in an attractor basin, as shown in S5 Fig. In this case, each colored dot represents a specific configuration of the network, with red dots representing configurations that converge to a single endpoint, in this case, the “Drug_Resistance” phenotype. This occurs because, during the simulation, the configurations progressively evolve until reaching a steady state or a limit cycle.

### Importance of complexity reduction

The main objective of this study is the reconstruction and reduction of BNs, in this case applied to the study of drug resistance in NSCLC. The aim is to simplify the network structure without losing the essence of its dynamic behavior, in order to find the minimal network that maintains the initial dynamics, assess its robustness, and determine if it has biological significance as a bioinformatic tool for identifying candidate genes.

Once it was confirmed that the rules for each node have biological grounding, the first reduction of the network was carried out, with the goal of simplifying the structure while preserving the essential dynamics that determine key phenotypes. Therefore, an analysis in terms of biological functions was conducted, leading to the removal of the nodes Sirt_1 and p53_INP1. The function of Sirt_1 is as a histone deacetylase that regulates various cellular processes, such as aging, inflammation, and stress response [28]. In cancer, its function is ambivalent: on one hand, it can act as an oncogene by promoting cell proliferation and oxidative stress resistance in tumor cells; on the other hand, in certain contexts, it also acts as a tumor suppressor by reducing inflammation and protecting DNA from damage [29]. As for p53_INP1 (Tumor protein p53 inducible nuclear protein 1), it is a stress-induced gene mainly regulated by p53. It promotes phosphorylation of p53 at serine 46, which increases its stability and transcriptional activity, crucial for activating genes such as p21 and PIG3, which halt cell growth and promote apoptosis in response to DNA damage [30].

Although both Sirt_1 and p53_INP1 have important roles in cancer in general, their roles in this network are redundant. Reviewing the rules in S1 Table in S1 File, Sirt_1 is activated when E2F1 is active and miR_145 and HDAC1 are inactive. Its main function is to regulate the nodes p53_A and p53_K, indirectly affecting p53, which influences network dynamics. However, p53 activation depends on multiple pathways, including ATM, KLF4, and Mdm2, which provide sufficient regulation to maintain the same steady states without the need for Sirt_1. Since Sirt_1 only has a secondary influence on p53 activation, its removal does not change the network’s behavior regarding the activation of terminal phenotypes.

For p53-INP1, it is a node activated when any form of p53 (p53_A or p53_K) is active, acting as an aggregator of p53 activity. Although p53-INP1 plays a role in activating p53_A and regulating Wip1, this function is redundant, as p53 alone can activate p53_A and other nodes. Additionally, Wip1 is also directly regulated by p53_A, allowing Wip1’s function to be maintained without the need for p53-INP1. Therefore, the removal of p53-INP1 does not affect the network’s ability to properly activate Wip1 or to maintain p53 regulation.

In this first elimination of these 2 nodes, the network was simplified, adapting the rules of the original 31-node network. These new rules are shown in S5 Table in S1 File. This resulted in a 29-node network that maintains the same steady states (“Proliferation,” “Drug_Resistance,” “Senescence,” and “Apoptosis”) and reduces the number of limit cycles to one of length 2, without altering the final phenotypes, as shown in Table 1. For this simulation, the terminal nodes were not considered, as they do not contribute to the overall dynamics; thus, 25 nodes were analyzed, and the phenotypes or terminal nodes were manually verified according to the rules. Comparing the configurations of each remaining node with the 31-node network showed no changes.

On the other hand, the network reduction shows that the nodes Sirt_1 and p53_INP1 sustain negative feedback loops (circuits with an odd number of inhibitions) capable of generating a limit cycle of period 4. Suppressing either of these nodes dismantles all negative cycles, causing the network dynamics to converge exclusively toward biologically relevant steady states. In regulatory network theory, a negative cycle is a closed path in which the signal undergoes an odd number of negations, such that an initial increase in a node eventually feeds back as an inhibition upon itself after a full loop. This delayed self-inhibition can lead to sustained oscillations, provided that the internal delay is sufficient. This criterion stems from the “Thomas conditions” [31], which state that the existence of at least one negative circuit is a necessary (but not sufficient) condition for the emergence of periodic behavior, whereas positive circuits are associated with multistability. Therefore, the removal of Sirt_1 or p53_INP1 eliminates this structural source of oscillations, resulting in the disappearance of spurious limit cycles of length 4.

The reduction process carried out in this study demonstrates the feasibility of simplifying complex networks without compromising the essential dynamics that define the phenotypes observed in NSCLC. The removal of redundant nodes, such as Sirt_1 and p53_INP1, highlights the importance of identifying components that, although biologically relevant, do not significantly contribute to the final configurations of the network. This strategy not only facilitates computational analysis by reducing the configuration space but also can improve our understanding of the key interactions underlying cellular phenotypes. The consistency of steady states and the length-two limit cycle in the 31-, 29-, 14-, and 9-node networks validates the robustness of this approach.

#### Generalizability of the reduction pipeline.

The four-step pipeline formalized in Network reduction pipeline combines steps that are fully algorithmic with steps that require manual biological curation. Specifically, fixing an input node (step 2), detecting nodes with fixed configurations (step 3), and identifying zero-out-degree terminal groups (step 4) can be automated for any Boolean network of comparable size, using standard graph-theoretic and attractor-computation algorithms. In contrast, the single-node deletion test (step 1) is algorithmic in its execution but requires biological judgment to decide *which* node removals are acceptable beyond the purely dynamical criterion. Similarly, the decision of which terminal groups to prune versus retain (step 4) may involve domain knowledge. We therefore expect steps 2–3 to be directly transferable to other Boolean models, while steps 1 and 4 will require case-by-case adaptation.

### Robustness of phenotypes against update schemes

The analysis of deterministic asynchronous update schemes in the minimal 9-node network provides additional evidence of model stability. In BNs, a synchronous simulation updates all nodes simultaneously at each time step, whereas deterministic asynchronous or block-sequential schemes update nodes or groups of nodes according to a prescribed order. Because biological regulatory processes do not necessarily occur simultaneously, exploring these alternative update schemes tests whether the phenotypes are artifacts of the synchronous assumption or remain stable under different relative timings [32,33].

As defined in Update digraphs and different dynamics in BNs, the representative schemes used in this analysis correspond to one representative per update-digraph equivalence class. Thus, they cover all distinct deterministic block-sequential dynamics induced by the dependency graph, rather than an arbitrary subset of possible schedules. In the original minimal 9-node network, this procedure identified 10,632 representative schemes; after rule fitting, the number increased to 23,107 representatives. The raw files supporting this comparison are provided as S2–S7 Files.

This increase is a topological and combinatorial consequence of the fitted BMI1 rule, not an increase in the number of network states. In the original 9-node network, BMI1 depends only on E2F1, whereas after fitting BMI1 depends on E2F1, p53_A, and p53_K. Therefore, two additional regulatory arcs, p53_A → BMI1 and p53_K → BMI1, are introduced. Since update-digraph equivalence labels each regulatory arc according to the relative update order of its source and target, these additional arcs refine the equivalence relation and split several previously equivalent schedules into distinct update-digraph classes. Both networks still contain 9 nodes and therefore the same 2^9^ = 512 possible configurations.

Importantly, the larger number of representative schemes in the fitted network does not indicate reduced robustness. On the contrary, the fitted rule increases dynamical robustness: the proportion of update-digraph classes yielding exclusively the three biologically relevant steady states increases from 69.19% to 94.59%, while the fraction of classes producing limit cycles decreases from 30.81% to 5.41%. The persistence of the drug-resistance, senescence, and apoptosis attractors across these representative schemes supports the conclusion that the minimal regulatory core is stable to changes in update timing.

It is particularly notable that the “Drug Resistance” phenotype exhibits the largest attractor basin in both synchronous and asynchronous simulations. This result coherently reflects the clinical reality, where drug resistance is a prevalent phenomenon in NSCLC patients [24]. The congruence between computational simulations and clinical observations suggests that the remaining nodes in the minimal network adequately capture the main determinants of this phenotype.

### Biological relevance of minimal network components

In analyzing the roles of these 9 nodes, a search was conducted for each component of the minimal network to understand their associations with NSCLC and cancer. It was found that:

- miR-145 (microRNA-145-5p): This microRNA acts as a tumor suppressor in NSCLC by regulating cell migration and invasion. Its decreased expression is associated with increased chemotherapy resistance due to interaction with Sp1 and BMI1, both related to the epithelial–mesenchymal transition (EMT) process [27,34].
- BMI1 (B lymphoma Mo-MLV insertion region 1 homolog): As part of the PRC1 complex, BMI1 is associated with epigenetic regulation in NSCLC and promotes tumor cell invasion and proliferation by inhibiting the expression of tumor suppressor genes. High BMI1 expression is associated with a worse prognosis in NSCLC patients [35].
- Sp1 (Specificity protein 1): This transcription factor is involved in regulating key genes for cell proliferation and has been shown to collaborate with BMI1 to induce treatment resistance in NSCLC. Inhibiting Sp1 could help restore chemotherapy sensitivity [27].
- MALAT1 (Metastasis-associated lung adenocarcinoma transcript 1): This long non-coding RNA (lncRNA) is implicated in promoting tumor growth in NSCLC by inhibiting microRNA-613 and increasing the expression of COMMD8, which promotes cell proliferation and reduces apoptosis. Its activity is therefore crucial in regulating tumor progression [36].
- E2F1 (E2F transcription factor 1): This transcription factor regulates genes involved in cell cycle progression. Its inappropriate activation is associated with increased proliferation and cell migration in NSCLC, contributing to tumor aggressiveness [37].
- p53 (Tumor suppressor p53 protein): This tumor suppressor gene is essential in regulating the cell cycle and apoptosis. Its inactivation or mutation facilitates tumor proliferation in NSCLC, and increasing its activity through p53 restoration could inhibit tumor growth [38,39].
- p53_A (Tumor suppressor p53 protein Ser-15 and Ser-20): is crucial for its activation in response to DNA damage, facilitated by the ATM kinase, promoting the tumor suppressor function of p53 by regulating genes that induce apoptosis [40]. Phosphorylation of p53 at Ser-15 and Ser-20 in A549 lung cancer cells exposed to cisplatin and air pollutants demonstrates that these sites are specifically activated in response to DNA damage, contributing to cell cycle arrest and controlled cell damage [41].
- p53_K (Tumor suppressor p53 protein Ser-46): Inhibition of p53 phosphorylation at Ser-46, induced by the drug rapamycin, has been shown to reduce its apoptotic activity in A549 lung cancer cells exposed to actinomycin D. This suggests that phosphorylation at Ser-46 is crucial for maintaining p53’s role in DNA damage-induced apoptosis [42].
- KLF4 (Krüppel-like Factor 4): KLF4 is a transcription factor that acts as a tumor suppressor and regulates cell proliferation and differentiation in NSCLC. Its reduced expression, promoted by specific microRNAs such as miR-3120-5p, is associated with increased metastatic potential [43]. Although it is active in the case of “Drug_resistance,” it depends on various genes and proteins, including p21, to regulate its function in cancer. KLF4 regulates this cell cycle inhibitor, which is essential for growth control and cell cycle progression, especially in its role as a tumor suppressor in different cancer contexts [44,45]. Observing the state of p21 in the 31-, 29-, and 14-node networks, in the “Drug Resistance” phenotype, it is inactive, indicating biological concordance of these nodes in this minimal network in generating the 3 phenotypes. Thus, this minimal network reflects the behavior of these genes concerning their phenotypes in NSCLC.

#### Attractor configurations and their biological interpretation.

To make the link between node states and phenotypes explicit, we describe the ON/OFF pattern for each of the three clinically relevant steady states (see Table 2):

- **Drug resistance** (steady state 1): characterized by miR_145  =  OFF, p53  =  OFF, p53_A  =  OFF, p53_K  =  OFF, and Sp1  =  ON, MALAT1  =  ON, BMI1  =  ON, KLF4  =  ON, E2F1  =  ON. This pattern reflects the loss of the tumor-suppressive miR-145/p53 axis and the concomitant activation of oncogenic drivers (MALAT1-mediated proliferation, E2F1-driven cell-cycle progression, and BMI1-mediated epigenetic silencing of tumor suppressors), consistent with the aggressive, therapy-resistant phenotype reported in NSCLC [27,35,36].
- **Senescence** (steady state 2): miR_145  =  ON, p53  =  ON, p53_K  =  ON (Ser-46 phosphorylation), p53_A  =  OFF, with Sp1, MALAT1, BMI1, KLF4, E2F1 all OFF. The activation of p53 and its Ser-46 isoform, together with the silencing of proliferative signals, is consistent with p53-mediated growth arrest and the induction of cellular senescence [38,42].
- **Apoptosis** (steady state 3): miR_145  =  ON, p53  =  ON, p53_A  =  ON (Ser-15/Ser-20 phosphorylation), p53_K  =  OFF, with Sp1, MALAT1, BMI1, KLF4, E2F1 all OFF. Activation of p53_A (Ser-15/Ser-20) is known to promote the pro-apoptotic transcriptional program of p53, activating downstream targets such as BAX and PUMA (present in the larger networks) [40,41]. The mutual exclusivity between p53_A-ON/p53_K-OFF (apoptosis) and p53_A-OFF/p53_K-ON (senescence) captures the experimentally documented phosphorylation-dependent switch between these two fates.

#### In silico perturbation examples.

To illustrate how the reduced and fitted networks can serve as hypothesis-generation tools, we evaluated a combined in silico perturbation on the synchronous attractor landscape of the fitted 9-node network. Rather than interpreting the nodes as isolated interventions, this analysis asks whether a coordinated perturbation of the miR-145/p53 axis and BMI1 is sufficient to redirect the network toward a specific cell-fate attractor.

Specifically, we fixed miR_145 and p53_A in the ON state and BMI1 in the OFF state (miR_145  =  1, p53_A  =  1, BMI1  =  0). Under this combined perturbation, all 64 compatible initial configurations converged to a single attractor: miR_145  =  ON, p53  =  ON, p53_A  =  ON, BMI1  =  OFF, with Sp1, MALAT1, KLF4, p53_K, and E2F1 OFF. This attractor corresponds to the apoptosis phenotype described above, because it combines activation of the tumor-suppressive miR-145/p53 module with the Ser-15/Ser-20 phosphorylated p53 state and loss of BMI1-mediated pro-survival regulation. The complete attractor configuration and basin size are reported in S16 Table in S1 File.

This result should be interpreted as a model-based hypothesis that requires experimental validation. Nevertheless, it shows that the minimal fitted network can prioritize combinatorial interventions and makes explicit that, in this case, the apoptotic response emerges from the joint perturbation of miR_145, p53_A, and BMI1 rather than from a single-node change alone.

### Boolean rule fitting for enhanced network dynamics

In the context of BNs applied to biology, an attractor is a set of states toward which a dynamical system tends, commonly representing stable cellular states such as differentiated cell types. These attractors are fundamental for modeling the stable behavior of cells and their fate in processes such as differentiation, proliferation, or apoptosis [10,46]. However, in complex computational models, what some authors conceptualize as spurious attractors may arise, these are steady states or limit cycles that do not correspond to real biological behaviors [47]. Such attractors are often the result of model limitations, poorly defined transition functions, or noisy or incomplete input data. These configurations can significantly affect the interpretation of the model, leading to false predictions about the system’s stability or its response to perturbations. To mitigate this problem, approaches such as the constrained genetic algorithm (CGA-BNI) have been developed; using steady-state gene-expression data, it imposes path-consistency constraints and selects BNs whose attractors more faithfully match the observed states, demonstrating a significant improvement in both dynamic and structural accuracy compared with other existing methods [48].

In our case, we developed an exhaustive and optimized fitting method aimed at generating and validating alternative Boolean rules in a gene regulatory network, with the goal of preserving only the attractors of interest. Starting from an initial set of rules, the method simulates the network’s steady states and then replaces each rule with new logical combinations constructed from other genes. For each gene, it evaluates whether these candidate rules are functionally equivalent in the steady states and, most importantly, whether they reproduce exactly the same set of desired attractors and not spurious ones. In this specific case, the original network produces three steady states and one spurious limit cycle (i.e., mixing different types of undesired configurations). Therefore, the code is designed to identify an alternative dynamic that eliminates the spurious cycle while retaining only the three relevant attractors.

The Boolean Rule Fitting Algorithm operates under the principle that for each target node, the algorithm explores increasingly complex combinations of possible regulatory nodes, generating candidate Boolean expressions using logical operators (AND, OR, NOT). Each candidate rule is evaluated on two levels: first, its local functionality is checked, that is, whether it correctly reproduces the value of the node in the original attractors. If it passes this test, its global consistency is then assessed by simulating the full network dynamics under the new rule to determine whether the resulting attractors match exactly the desired ones.

To generate rules, logical combinations were explored that involve one, two or three potential regulators of the target node, applying a construction logic based on standard Boolean operators: negation (!), conjunction (&), and disjunction (|). In the case of a single regulatory variable, the set of candidate expressions was limited to two possible forms: the variable in its direct form and its negation. For two variables, all possible combinations were generated using the mentioned operators, including variants with one or both variables negated. This yielded a total of eight configurations per pair, such as [X & Y], [!X & Y], [X | !Y], [!X | !Y], etc., covering a basic yet expressive range of binary logical relationships.

For combinations involving three regulators, a broader strategy was adopted that included expressions without parentheses and expressions with explicit parentheses. The parenthesis-free expressions consisted of direct combinations of the three terms, evaluated according to standard logical precedence (where “!” has higher priority than “&,” and “&” has higher priority than “|”). Typical examples include expressions such as [X & Y & Z], [!X | Y | Z], [!X & !Y & Z], etc. Although syntactically simple, these forms capture multiple modes of simultaneous activation and repression among regulators.

On the other hand, parenthesized expressions were used to represent more complex hierarchical or conditional interactions, where two inputs are logically grouped before interacting with a third. Examples of these structures include [(!X & Y) | Z], [(X | !Y) & !Z], [(X & Y) | !Z], etc. This type of construction reflects common patterns in gene regulation, where certain effects manifest only under specific combinations of signals, adding realism to the logical model.

This approach enables exploration of a broad expressive space while maintaining an interpretable and biologically plausible logical structure. The diversity of generated forms ensures that both simple rules and complex interactions are considered, thereby facilitating the identification of alternative rules that preserve the desired dynamics of the network while eliminating spurious behaviors inconsistent with the biological framework. Rule generation was limited to combinations of up to three regulators, according to the Kauffman K parameter, which was set to a maximum of 3.

Kauffman’s Boolean network parameter K controls connectivity and has a crucial impact on system behavior: When K is low, the system is ordered; when K is high, it becomes chaotic. Therefore, there exists a critical value Kc that marks the transition between order and chaos.

Classical studies, such as those by Derrida [49], estimated this critical value at Kc = 2 for unbiased Boolean functions. However, investigations like those by Zertuche have corrected this value by taking into account Boolean irreducibility, a structural property of the functions used. With this correction, the critical value is adjusted to $K_{c}\approx 2.62$ [50].

Networks with *K* > 3 are in the chaotic phase, characterized by high sensitivity to initial conditions, a large number of attractors, and loss of system stability. This behavior has been confirmed through simulations and analyses in different network topologies, including scale-free networks and generalized models [51,52]. In our rule-fitting algorithm, the restriction to candidate rules with at most *K* = 3 regulators is a direct design choice informed by this critical-connectivity theory: by keeping the in-degree of any fitted rule at or below 3, we ensure that the modified network remains in the ordered or near-critical regime ($K\leq K_{c}\approx 2.62$), avoiding the chaotic phase and preserving the stability properties that make the model biologically interpretable.

#### Scalability.

In principle, the rule-fitting algorithm could be applied to the original 31-node network. In practice, however, the exhaustive enumeration of candidate Boolean expressions with up to *K* = 3 regulators drawn from 31 nodes generates (30k) combinations for each k∈{1,2,3}, and each candidate requires a full simulation of the $2^{31}\approx 2\times 10^{9}$-state transition graph; for further details on the underlying combinatorial formulas, see [23]. At the 9-node scale, exhaustive evaluation runs in minutes; at 31 nodes, the number of configurations alone makes brute-force simulation prohibitive without substantial additional pruning, heuristic search, or symbolic techniques (e.g., binary decision diagrams). Extending the approach to larger networks would thus require incorporating one or more of these strategies, which lies beyond the scope of the present work.

The exhaustive search returned 52 dynamically admissible substitutions across the nine target nodes. A subsequent biological review prioritized five candidates (BMI1 at rank 1, MALAT1 at rank 2, miR-145 at rank 3, and two p53 alternatives at ranks 4 and 5) while the BMI1 rule was carried forward for the fitted-network analyses. The selected BMI1 rule, “(!p53_A&!p53_K)|E2F1”, combines two logical branches. Its E2F1 term is consistent with promoter-level evidence that BMI1 is a direct transcriptional target of E2F1, although this interaction was established in neuroblastoma rather than NSCLC [25]. The !p53_A&!p53_K term is interpreted as p53-state-dependent BMI1 derepression when both phosphorylated p53 nodes are inactive, rather than as a direct interaction between phosphorylated p53 and the BMI1 promoter. Because p53 transcriptional outputs are highly context dependent [53], evidence from other cancer systems should be treated as mechanistic support rather than NSCLC-specific validation. In mammary epithelial and breast cancer models, p53 was shown to transcriptionally activate miR-200c, and loss of this pathway was associated with increased BMI1 expression [26]. In NSCLC, independent experimental evidence links the BMI1/miR-145-5p/Sp1 axis to EMT and pemetrexed resistance [27]. Thus, the selected rule combines an experimentally supported E2F1 activation term with a biologically plausible p53-state-dependent constraint whose specific implementation in NSCLC remains a model-derived hypothesis requiring experimental validation. The other four shortlisted rules remain dynamically valid and biologically plausible alternatives, but they were not substituted into the final fitted network; their biological rationales are summarized in S11 Table in S1 File.

Subsequently, after simulating the modified rule and confirming that the original steady states remained unchanged, this adjustment was applied to the 29-node network. As in the reduced nine-node version, the length-two limit cycle was abolished and the steady-state configurations remained intact. Finally, upon evaluating asynchronous update schemes and comparing the 9 node network before and after this modification, the proportion of schemes yielding exclusively steady states increased from 69.19% to 94.59%, thereby markedly reducing the number of schemes that produced spurious cycles.

### Therapeutic implications

#### Qualitative comparison with clinical response frequencies.

A recurring observation across all reduced networks is that the basin of attraction associated with the “Drug Resistance” phenotype is markedly larger than those of senescence and apoptosis. Clinical studies of cisplatin  +  pemetrexed first-line therapy in advanced NSCLC consistently report objective response rates in the range of approximately 20%−30% [24], implying that roughly 70%−80% of treated patients do not exhibit a favorable response and are de facto classified as resistant. The basin sizes obtained in our simulations for the same combination context are summarized in Table 3: across all reduced networks (14-, and 9-node) and both synchronous and asynchronous update regimes, the “Drug Resistance” basin dominates. After rule fitting, this proportion further increases to 99.22% under synchronous update.

**Table 3:** Qualitative comparison between attraction basin sizes (%) of the “Drug Resistance” steady state across reduced networks/update regimes and reported clinical response frequencies for cisplatin  +  pemetrexed in NSCLC. Values for synchronous simulations are exact (exhaustive enumeration); asynchronous values are aggregated across all representative deterministic update schemes (see S9 and S14 Tables in S1 File). The agreement is qualitative, not quantitatively calibrated.

Setting	Resistance basin (%)	Non-resistance basin (%)
14-node, synchronous	98.44	1.56
9-node, synchronous	98.44	1.56
9-node, fitted, synchronous	99.22	0.78
9-node, asynchronous (avg.)	~85	~15
9-node, fitted, asynchronous (avg.)	~90	~10
Clinical (cisplatin+pemetrexed) [24]	~70 – 80 (non-response)	~20 – 30 (response)

We emphasize that this comparison is *qualitative*, not quantitatively calibrated: clinical response is influenced by patient-specific factors (mutational status, EGFR/ALK alterations, comorbidities, tumor stage) that are not represented in the model, and the basin proportions assume a uniform distribution of initial configurations, which need not match the actual distribution of cellular states in tumors. A quantitative alignment between model basins and clinical frequencies would require, at a minimum: (i) an explicit prior over initial states informed by single-cell or bulk-expression data; (ii) inclusion of patient-level mutation patterns and tumor heterogeneity; and (iii) calibration against curated clinical cohorts. Such a calibration lies beyond the scope of the present work, whose goal is to establish the dynamical and structural soundness of the minimal model.

#### Candidate biomarkers and hypothesis-generating targets.

With these caveats in mind, the dominance of the resistance attractor and the structural simplicity of the 9-node core suggest that the corresponding components (miR_145, Sp1, MALAT1, BMI1, KLF4, p53, p53_A, p53_K, and E2F1) are reasonable *candidate biomarkers* of cisplatin/pemetrexed resistance, and that perturbations targeting them could be explored as *hypothesis-generating* therapeutic strategies aimed at promoting the senescence or apoptosis attractors. Any clinical use of these predictions would require dedicated experimental validation.

The identification of attractor configurations associated with undesirable phenotypes, such as drug resistance, also opens the possibility of designing genetic or pharmacological interventions aimed at modifying these configurations. In this sense, the simplified model could serve as a useful tool for exploring therapeutic strategies computationally before experimental validation. Rules that satisfy both criteria are considered valid. At the end of the process, an alternative set of rules is generated for each node, reproducing the expected dynamics without introducing spurious behaviors.

### Model limitations

Despite the dynamical and biological coherence of the proposed minimal model, several assumptions limit the realism of the present analysis and should be made explicit. The Boolean formalism assigns a single binary state (0/1) to each gene, microRNA, or lncRNA, thereby collapsing graded expression levels and post-translational regulation into a coarse on/off abstraction. Moreover, neither synchronous nor asynchronous updating represents the physical time scales of transcription, translation, or degradation; these schemes encode only the relative ordering of regulatory events.

At the cellular level, the model represents an “average” tumor cell and therefore does not capture intratumoral heterogeneity, clonal evolution, or microenvironmental effects. In addition, the 14- and 9-node reductions were derived under the constraint DNA_Damage = 1, so they characterize regulatory dynamics during *persistent* drug-induced damage but are not designed to describe damage initiation, DNA repair, or drug withdrawal. All update schemes examined here are deterministic; incorporating stochastic asynchronous updates or noise in the regulatory functions could alter basin sizes and help refine future quantitative predictions.

Finally, both the network topology and the original Boolean rules were inherited from a single published source [2], with manually curated adjustments. Alternative network reconstructions or rule sets could yield different basin distributions. Taken together, these limitations mean that the basin sizes reported here should be interpreted as *qualitative* indicators of phenotype propensity rather than as quantitative predictions of clinical response frequencies.

## Conclusions

Our results demonstrate that aggressive down-scaling of a biologically grounded Boolean network can be achieved without sacrificing dynamical fidelity. Starting from a 31-node model of cisplatin- and pemetrexed-resistance in NSCLC, we constructed a minimal 9-node network that exactly preserves the attractor landscape and reproduces three clinically relevant steady states together with their basins of attraction. Intermediate 29- and 14-node models show that the reduction procedure is gradual and biologically interpretable, highlighting redundant feedback loops and terminal nodes that do not contribute to the core resistance mechanisms.

By combining update-digraph theory with exhaustive simulations, we showed that the three phenotypes of interest are robust across more than ten thousand deterministic update schemes of the minimal network. Furthermore, an exhaustive Boolean rule-fitting algorithm allowed us to remove spurious limit cycles while preserving the same set of attractors, substantially increasing the proportion of update schemes that yield only steady states. Together, these findings indicate that a small regulatory core involving miR-145, MALAT1, BMI1, Sp1, KLF4, E2F1 and phosphorylated p53 isoforms is sufficient to encode the observed drug-resistance behavior.

From a biological and translational perspective, the reduced model provides a tractable scaffold for in silico perturbation analyses and for prioritizing candidate biomarkers or therapeutic targets related to chemoresistance in NSCLC. Future work should integrate time-series data and additional molecular players to refine the regulatory rules, and experimentally validate the predicted impact of perturbing the identified master regulators on drug-response phenotypes.

## Supporting information

S1 Fig The 31-node Boolean network for NSCLC. The network was reconstructed from the one that appears in [2], where green arcs represent positive weights (activations), and red arcs represent negative weights (inhibitions). The red-colored node represents “DNA_Damage”, which corresponds to the input node; the four phenotypes that can be generated in this network are shown in pink, and the biological components corresponding to long non-coding RNAs, microRNAs, and various transcription factors are in blue.
(TIFF)

S2 FigUpdate digraphs associated with the digraph *G* of Example 1.Panels I–IX correspond to representative schedules $s_{1},...,s_{9}$; panel VI is also obtained with *s*_10_ and *s*_13_, and panel VIII is also obtained with *s*_11_ and *s*_12_.(TIFF)

S3 FigAll the distinct dynamics of S3 Table seen as state transition graphs for Example 1.Panels I–IX are associated with the representative schemes $s_{1},...,s_{9}$, respectively.(TIFF)

S4 Fig Reduced 14-node Boolean network for NSCLC. To construct this network, the 29-node network was simplified from the information in S6 Table in S1 File. The 29-node network was simplified for the case where “DNA_Damage” equals 1, discarding nodes that fixed their configuration similarly in steady states and limit cycles, and also eliminating terminal nodes. Green arcs represent positive weights (activations), and red arcs represent negative weights (inhibitions).
(TIFF)

S5 Fig Attraction basin for the synchronous simulation of the 9-node network. This figure presents the attraction basins for the synchronous simulation of the 9-node network. Red points represent the attraction basin for “Drug Resistance”, with a size of 504 configurations. Light blue points represent the attraction basin for “Senescence”, with a size of 2 configurations. Blue points represent the attraction basin for “Apoptosis”, with a size of 2 configurations. Green points represent the attraction basin for the limit cycle, with a size of 4 configurations.
(TIFF)

S1 File Supporting figures, tables, and algorithms. This file contains all supporting figures, tables, and algorithms presented with the manuscript: S1-S5 Figs, S1 Table - S16 Table [54–59], and Algorithms 1–4.
(PDF)

S2 File Representative deterministic update schemes for the original 9-node network. Each row corresponds to one representative update-digraph equivalence class for the original 9-node network. Columns list the scheme identifier and the update block assigned to each node (miR_145, Sp1, MALAT1, BMI1, KLF4, p53, p53_A, p53_K, and E2F1). This file contains the 10,632 representative deterministic update schemes used in Analysis of Update Schemes.
(CSV)

S3 File Raw attractor and basin-size results for the original 9-node network. For each representative scheme, the file reports the update vector, each detected attractor as a binary state or cycle, and the corresponding basin size. These values underlie the reported counts of schemes with only steady states, schemes with limit cycles, and the basin-size summaries for the original 9-node network.
(CSV)

S4 File Summary statistics for deterministic update schemes of the original 9-node network. This file summarizes the 10,632 representative schemes, including the numbers and percentages of schemes with only fixed points or with limit cycles, the repeated fixed points and their average basin sizes, the most frequent limit cycles, and the distribution of limit cycles by number and length.
(TXT)

S5 File Representative deterministic update schemes for the rule-fitted 9-node network. Each row corresponds to one representative update-digraph equivalence class for the rule-fitted 9-node network. Columns list the scheme identifier and the update block assigned to each node. This file contains the 23,107 representative deterministic update schemes used to evaluate the fitted network.
(CSV)

S6 File Raw attractor and basin-size results for the rule-fitted 9-node network. For each representative scheme, the file reports the update vector, each detected attractor as a binary state or cycle, and the corresponding basin size. These values underlie the reported robustness increase after rule fitting and the comparison between the original and fitted 9-node networks.
(CSV)

S7 File Summary statistics for deterministic update schemes of the rule-fitted 9-node network. This file summarizes the 23,107 representative schemes of the rule-fitted network, including the numbers and percentages of schemes with only fixed points or with limit cycles, repeated fixed points and their average basin sizes, the most frequent limit cycles, and the distribution of limit cycles by number and length.
(TXT)
