# Supplementary material for: Boolean-network simplification and rule fitting to unravel chemotherapy resistance in non-small cell lung cancer
Source: PLoS One. 2026 Sep 15;21(9):e0357764. doi: 10.1371/journal.pone.0357764 (PMC13577482; doi:10.1371/journal.pone.0357764)
Supplement: Supplementary file 1 [file pone.0357764.s006.pdf]

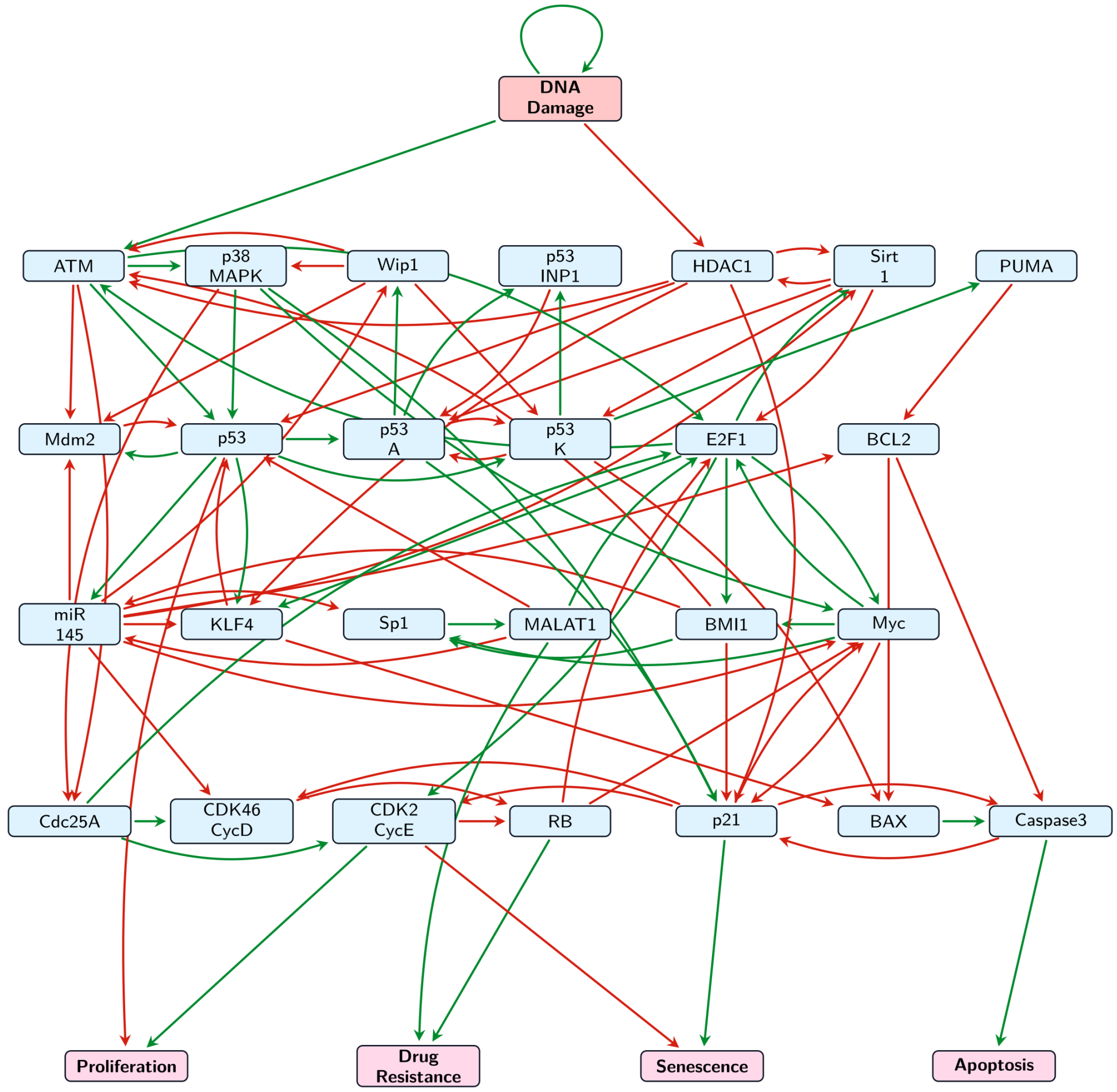

S1 Fig. The 31-node Boolean network for NSCLC reconstructed from the one that appears in [2], where green arcs represent positive weights (activations), and red arcs represent negative weights (inhibitions). The red-colored node represents "DNA_Damage", which corresponds to the input node; the four phenotypes that can be generated in this network are shown in pink, and the biological components corresponding to long non-coding RNAs, microRNAs, and various transcription factors are in blue.

S1 Table. The 31 local boolean functions (or interactions) associated to the nodes of the NSCLC network shown in S1 Fig.

Node	Interactions
ATM	DNA_Damage & (!HDAC1 | !Wip1 | E2F1 | !BMI1)
p38MAPK	ATM & !Wip1
miR_145	p53 & !MALAT1 & !BMI1
Sp1	(BMI1 & Myc) | !miR_145
MALAT1	Sp1
BMI1	Myc | E2F1
KLF4	!miR_145 | (E2F1 & !HDAC1 & p53)
HDAC1	!Sirt_1 & !DNA_Damage
Myc	(E2F1 | p38MAPK | !p21) & !RB & !miR_145
p53	(ATM & !KLF4) | (!Mdm2 & p38MAPK & !HDAC1 & !MALAT1)
Mdm2	(!Wip1 | p53) & !ATM & !miR_145
p53_A	!Sirt_1 & !p53_K & (p53 | !p53_INP1)
p53_K	!p53_A & (!Sirt_1 | !Wip1) & p53
Sirt_1	E2F1 & !miR_145 & !HDAC1
p53_INP1	p53_A | p53_K
Wip1	p53_A & !miR_145
p21	p53_A | ((!HDAC1 & !Myc) & !BMI1 & !Caspase3 & p38MAPK)
Cdc25A	!ATM & !p38MAPK
CDK46_CycD	Cdc25A & !miR_145 & !p21
CDK2_CycE	Cdc25A & E2F1 & !p21
RB	!CDK46_CycD & !CDK2_CycE
PUMA	p53_K
BCL2	!PUMA & !miR_145
BAX	(!BCL2 | !KLF4) & !p53_K
E2F1	(!RB & ((Cdc25A & ATM) | !Sirt_1)) | MALAT1 | Myc
Caspase3	(!BCL2 | !p21) & BAX
DNA_Damage	DNA_Damage
**Proliferation**	CDK2_CycE & !p53
**Drug_Resistance**	MALAT1 & RB
**Senescence**	p21 & !CDK2_CycE
**Apoptosis**	Caspase3

S2 Table. Equivalence classes of deterministic update schedules for the digraph in Example 1. Each column contains the schedules that produce the same update digraph and therefore the same deterministic dynamics. The representative schedules used in the example are s_i, $i = 1, \dots, 9$.

$[s_1]_G$	$[s_2]_G$	$[s_3]_G$	$[s_4]_G$	$[s_5]_G$	$[s_6]_G$	$[s_7]_G$	$[s_8]_G$	$[s_9]_G$
(A,B,C)	(B)(A,C)	(A,C)(B)	(B,C)(A)	(B)(C)(A)	(C)(A,B)<br>(C)(A)(B)<br>(C)(B)(A)	(A)(B,C)	(A,B)(C)<br>(A)(B)(C)<br>(B)(A)(C)	(A)(C)(B)

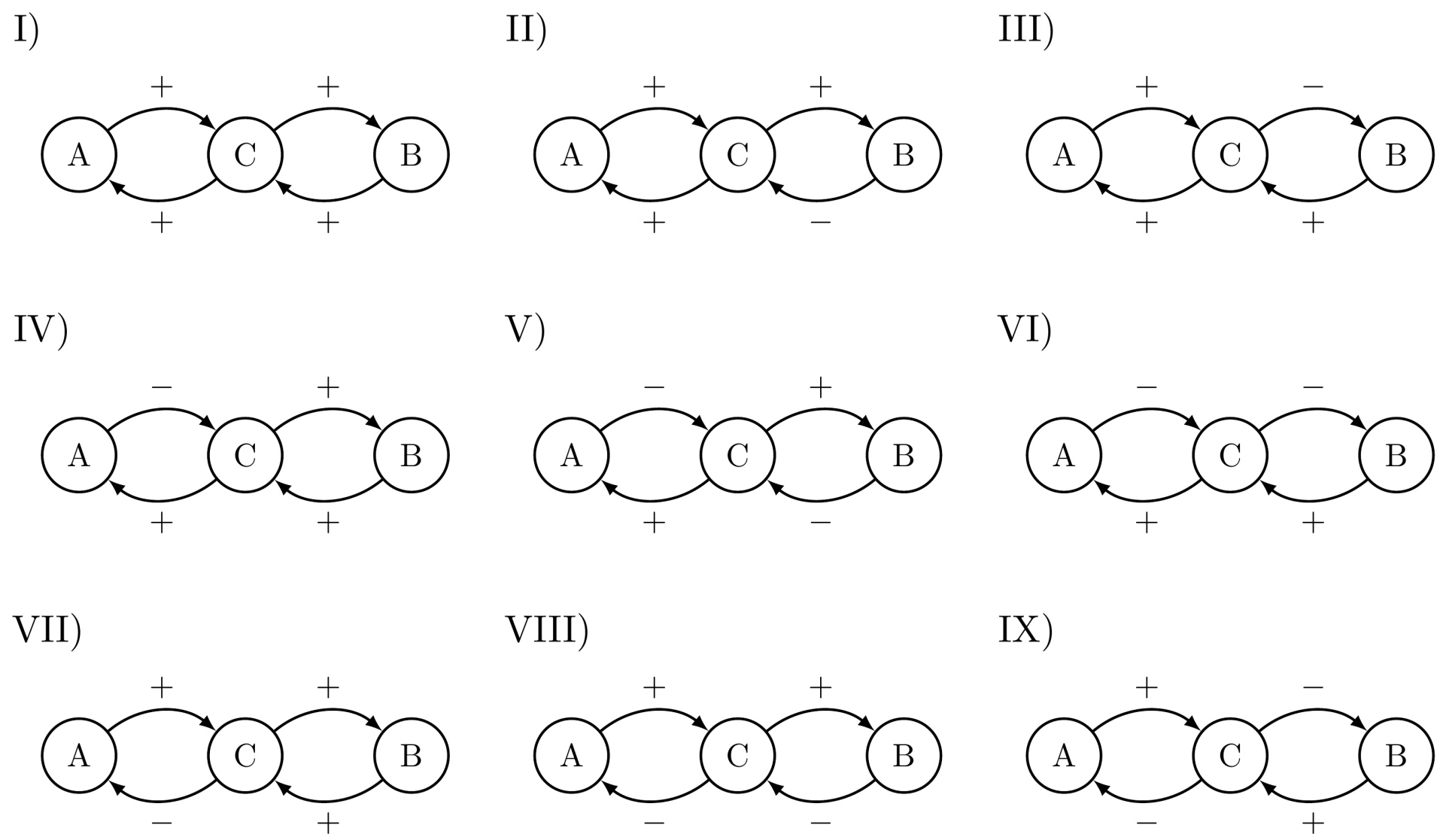

S2 Fig. Update digraphs associated with the digraph G of Example 1. Panels I–IX correspond to representative schedules $s_1, \ldots, s_9$; panel VI is also obtained with s_{10} and s_{13}, and panel VIII is also obtained with s_{11} and s_{12}.

S3 Table. All the distinct dynamics (seen as transition tables) that a BN with the digraph G and the Boolean local functions given in the Example 1 can have by using the (representative) schemes s_1, ..., s_9.

State	Sched. 1	Sched. 2	Sched. 3	Sched. 4	Sched. 5	Sched. 6	Sched. 7	Sched. 8	Sched. 9
	s_1(A)=1<br>s_1(B)=1<br>s_1(C)=1	s_2(A)=2<br>s_2(B)=1<br>s_2(C)=2	s_3(A)=1<br>s_3(B)=2<br>s_3(C)=1	s_4(A)=2<br>s_4(B)=1<br>s_4(C)=1	s_5(A)=3<br>s_5(B)=1<br>s_5(C)=2	s_6(A)=2<br>s_6(B)=2<br>s_6(C)=1	s_7(A)=1<br>s_7(B)=2<br>s_7(C)=2	s_8(A)=1<br>s_8(B)=1<br>s_8(C)=2	s_9(A)=1<br>s_9(B)=3<br>s_9(C)=2
000	000	000	000	000	000	000	000	000	000
001	110	110	100	010	010	000	110	111	100
010	000	000	000	000	000	000	000	000	000
011	110	110	100	010	010	000	111	111	111
100	000	000	000	000	000	000	000	000	000
101	110	111	100	010	111	000	110	111	100
110	001	000	011	101	000	111	000	000	000
111	111	111	111	111	111	111	111	111	111

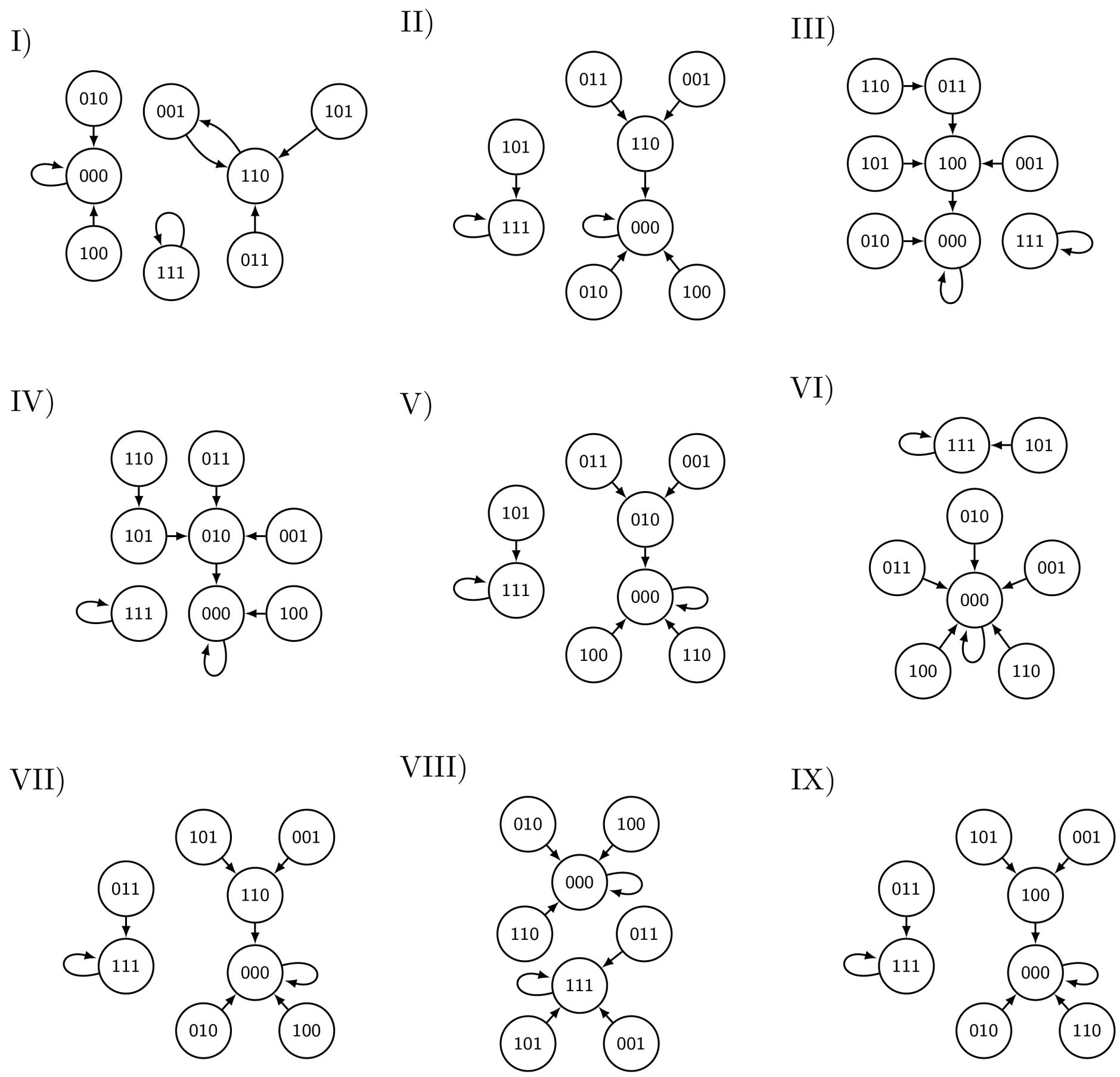

S3 Fig. All the distinct dynamics of S3 Table seen as state transition graphs for Example 1. Panels I–IX are associated with the representative schemes $s_1, \ldots, s_9$, respectively.

S4 Table. attractors results for the 31-node network simulation.

Components	SteadyState1	SteadyState2	SteadyState3	SteadyState4	LimitCycle1	LimitCycle2	LimitCycle3
ATM	0	1	1	1	1 1	0 0 0 0	0 0 0 0
p38MAPK	0	1	1	1	1 1	0 0 0 0	0 0 0 0
miR_145	0	0	1	1	1 1	0 0 0 0	0 0 0 0
Sp1	1	1	0	0	0 0	1 1 1 1	1 1 1 1
MALAT1	1	1	0	0	0 0	1 1 1 1	1 1 1 1
BMI1	1	1	0	0	0 0	1 1 1 1	1 1 1 1
KLF4	1	1	0	0	0 0	1 1 1 1	1 1 1 1
HDAC1	0	0	0	0	0 0	1 1 1 1	1 0 1 0
Myc	1	0	0	0	0 0	1 1 0 0	1 1 0 1
p53	0	0	1	1	1 1	0 0 0 0	0 0 0 0
Mdm2	1	0	0	0	0 0	0 0 1 1	0 1 1 1
p53_A	0	0	0	1	1 0	0 0 1 1	0 0 1 0
p53_K	0	0	1	0	1 0	0 0 0 0	0 0 0 0
Sirt_1	1	1	0	0	0 0	0 0 0 0	1 0 1 0
p53_INP1	0	0	1	1	0 1	1 0 0 1	0 0 0 1
Wip1	0	0	0	0	0 0	1 0 0 1	0 0 0 1
p21	0	0	1	1	0 1	1 0 0 1	0 0 0 1
Cdc25A	1	0	0	0	0 0	1 1 1 1	1 1 1 1
CDK46_CycD	1	0	0	0	0 0	0 0 1 1	0 1 1 1
CDK2_CycE	1	0	0	0	0 0	0 0 1 1	0 1 1 1
RB	0	1	1	1	1 1	0 1 1 0	0 1 0 0
PUMA	0	0	1	0	0 1	0 0 0 0	0 0 0 0
BCL2	1	1	0	0	0 0	1 1 1 1	1 1 1 1
BAX	0	0	0	1	1 0	0 0 0 0	0 0 0 0
E2F1	1	1	0	0	0 0	1 1 1 1	1 1 1 1
Caspase3	0	0	0	1	0 1	0 0 0 0	0 0 0 0
DNA_Damage	0	1	1	1	1 1	0 0 0 0	0 0 0 0
**Proliferation**	1	0	0	0	- -	- - - -	- - - -
**Drug Resistance**	0	1	0	0	- -	- - - -	- - - -
**Senescence**	0	0	1	1	- -	- - - -	- - - -
**Apoptosis**	0	0	0	1	- -	- - - -	- - - -

S5 Table. Adapted rules for the 29-node network.

Node	Interactions
ATM	DNA_Damage & (!HDAC1 | !Wip1 | E2F1 | !BMI1)
p38MAPK	ATM & !Wip1
miR_145	p53 & !MALAT1 & !BMI1
Sp1	(BMI1 & Myc) | !miR_145
MALAT1	Sp1
BMI1	Myc | E2F1
KLF4	!miR_145 | (E2F1 & !HDAC1 & p53)
HDAC1	!DNA_Damage
Myc	(E2F1 | p38MAPK | !p21) & !RB & !miR_145
p53	(ATM & !KLF4) | (!Mdm2 & p38MAPK & !HDAC1 & !MALAT1)
Mdm2	(!Wip1 | p53) & !ATM & !miR_145
p53_A	!p53_K & (p53)
p53_K	!p53_A & (!Wip1) & p53
Wip1	p53_A & !miR_145
p21	p53_A | ((!HDAC1 & !Myc) & !BMI1 & !Caspase3 & p38MAPK)
Cdc25A	!ATM & !p38MAPK
CDK46_CycD	Cdc25A & !miR_145 & !p21
CDK2_CycE	Cdc25A & E2F1 & !p21
RB	!CDK46_CycD & !CDK2_CycE
PUMA	p53_K
BCL2	!PUMA & !miR_145
BAX	(!BCL2 | !KLF4) & !p53_K
E2F1	(!RB & ((Cdc25A & ATM))) | MALAT1 | Myc
Caspase3	(!BCL2 | !p21) & BAX
DNA_Damage	DNA_Damage
**Proliferation**	CDK2_CycE & !p53
**Drug_Resistance**	MALAT1 & RB
**Senescence**	p21 & !CDK2_CycE
**Apoptosis**	Caspase3

S6 Table. Adapted rules for the 14-node network.

Node	Interactions
miR_145	p53 & !MALAT1 & !BMI1
Sp1	(BMI1) | !miR_145
MALAT1	Sp1
BMI1	E2F1
KLF4	!miR_145 | (E2F1 & p53)
p53	!KLF4 | !MALAT1
p53_A	!p53_K & p53
p53_K	!p53_A & p53
BAX	(!BCL2 | !KLF4) & !p53_K
E2F1	MALAT1
Caspase3	(!BCL2 | !p21) & BAX
p21	p53_A | (!BMI1 & !Caspase3)
BCL2	!PUMA & !miR_145
PUMA	p53_K

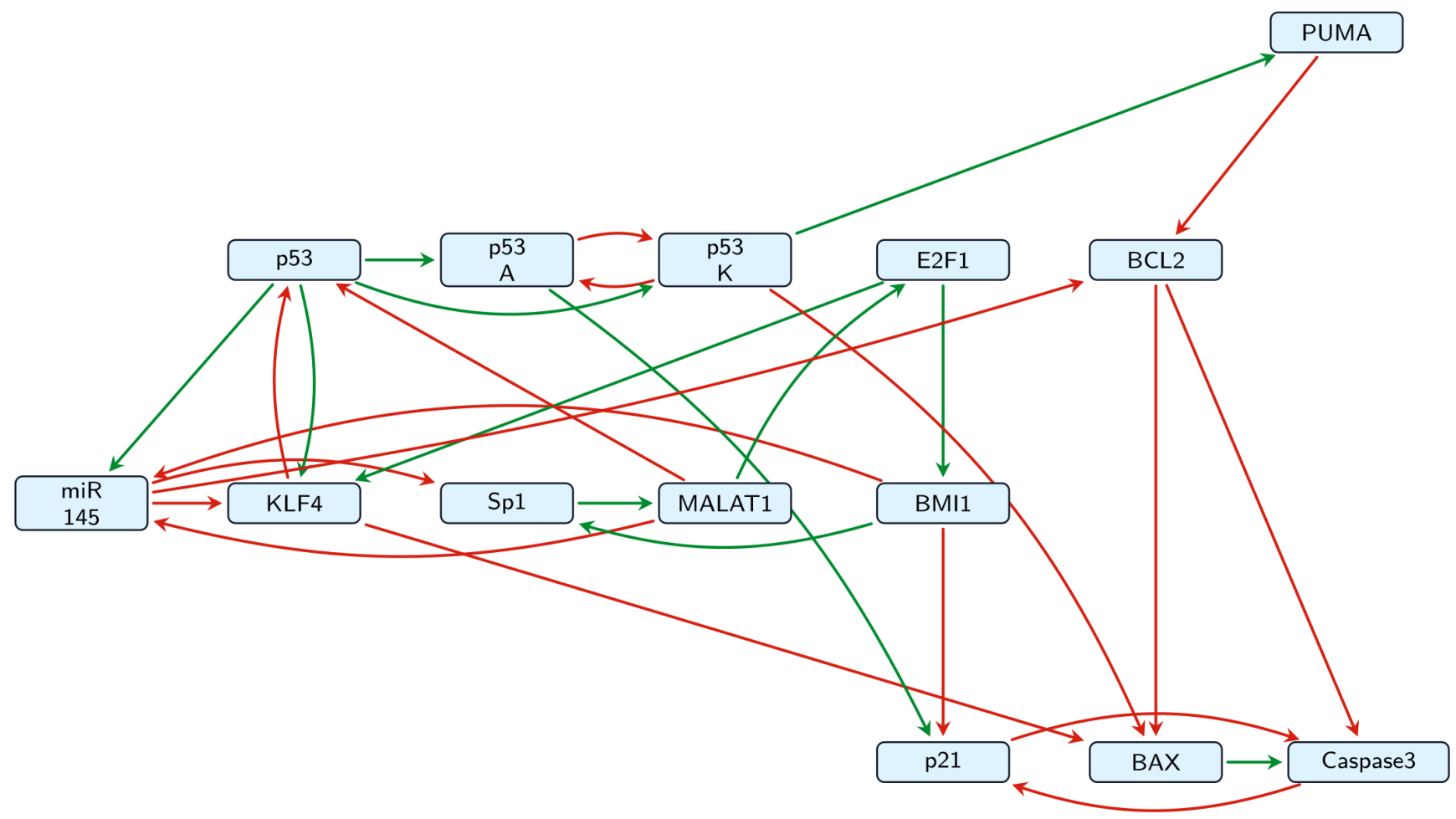

S4 Fig. Reduced 14-node Boolean network for NSCLC. To construct this network, the 29-node network was simplified from the information in S6 Table. The 29-node network was simplified for the case where "DNA_Damage" equals 1, discarding nodes that fixed their configuration similarly in steady states and limit cycles, and also eliminating terminal nodes. Green arcs represent positive weights (activations), and red arcs represent negative weights (inhibitions).

S7 Table. Attractors results for the 14-node network simulation.

Components	Steady state 1	Steady state 2	Steady state 3	Limit Cycle 1
miR_145	0	1	1	1 1
Sp1	1	0	0	0 0
MALAT1	1	0	0	0 0
BMI1	1	0	0	0 0
KLF4	1	0	0	0 0
p53	0	1	1	1 1
p53_A	0	0	1	1 0
p53_K	0	1	0	1 0
p21	0	1	1	0 1
PUMA	0	1	0	0 1
BCL2	1	0	0	0 0
BAX	0	0	1	1 0
E2F1	1	0	0	0 0
Caspase3	0	0	1	0 1
**Attraction basin**	98.44	0.39	0.39	0.78

S8 Table. Adapted rules for the 9-node network.

Node	Interactions
miR_145	p53 & !MALAT1 & !BMI1
Sp1	(BMI1) | !miR_145
MALAT1	Sp1
BMI1	E2F1
KLF4	!miR_145 | (E2F1 & p53)
p53	!KLF4 | !MALAT1
p53_A	!p53_K & p53
p53_K	!p53_A & p53
E2F1	MALAT1

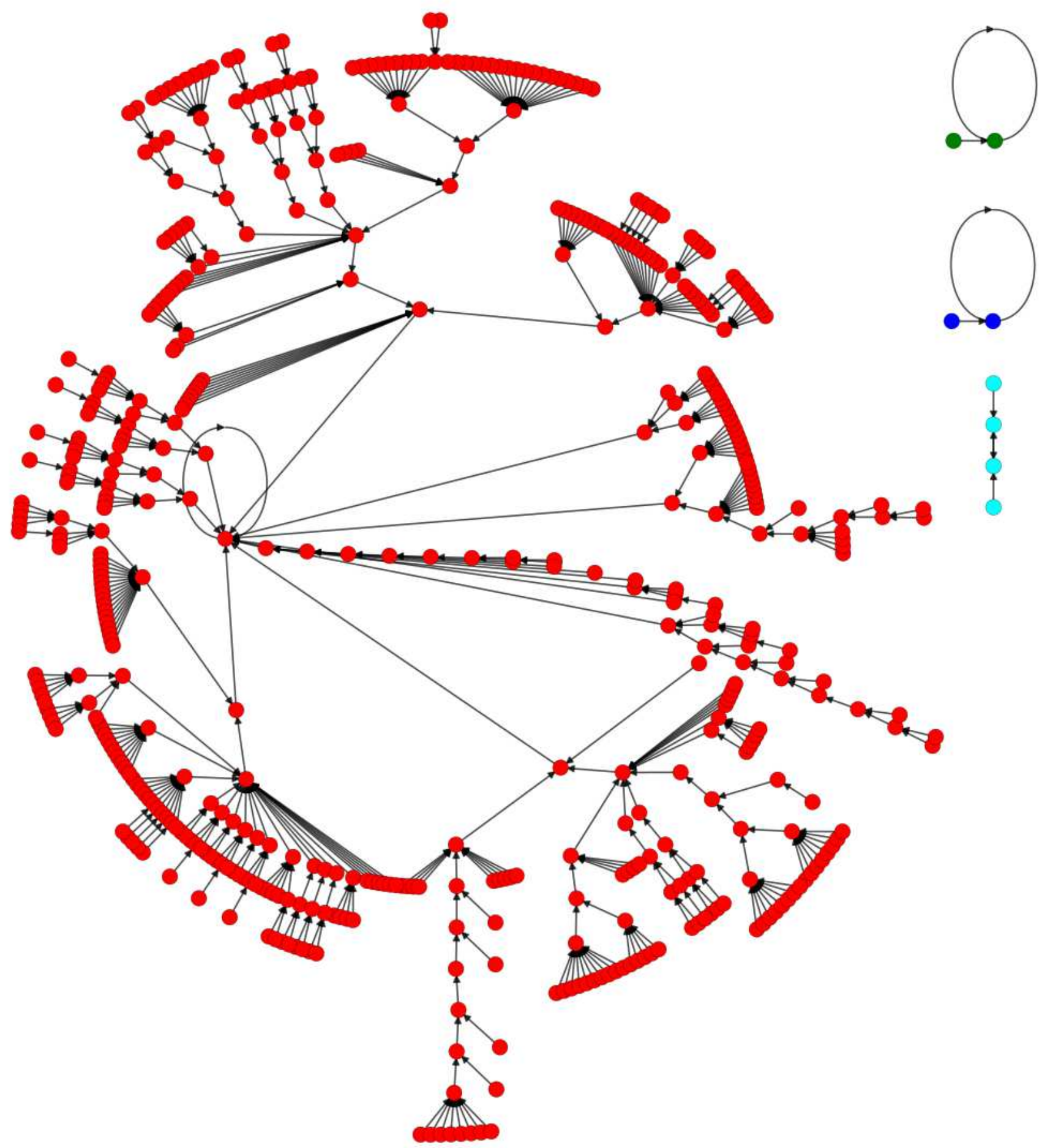

S5 Fig. Attraction basin for the synchronous simulation of the 9-node network. This figure presents the attraction basins for the synchronous simulation of the 9-node network. Red points represent the attraction basin for "Drug Resistance", with a size of 504 configurations. Light blue points represent the attraction basin for "Senescence", with a size of 2 configurations. Blue points represent the attraction basin for "Apoptosis", with a size of 2 configurations. Green points represent the attraction basin for the limit cycle, with a size of 4 configurations.

S9 Table. Steady state analysis across all representative asynchronous update schemes of the minimum 9-node network. "Average Basin Attraction" is the mean number of initial configurations (out of $2^9 = 512$) converging to each attractor, averaged over all representative schemes in which the attractor appears; "SD" is the standard deviation of basin sizes across those schemes; "Count" is the number of representative schemes in which the attractor is present.

Steady state Configuration	Average Basin Attraction	SD	Count
011110001	441.19	69.43	10,632
100001010	23.25	21.86	10,632
100001100	23.25	21.86	10,632

S10 Table. Top 10 most frequent limit cycles across all representative asynchronous update schemes for the minimum 9-node network. "Average Basin Attraction" is the mean number of initial configurations converging to the cycle, averaged over all representative schemes in which the cycle appears; "SD" is the corresponding standard deviation; "Count" is the number of representative schemes producing that cycle; "Percent" is the fraction of all cycle-producing schemes in which this particular cycle appears.

Limit cycle Configuration	Average Basin Attraction	SD	Count	Percent
100001000, 100001110	31.50	26.57	2,658	67.29
010111101, 101001100	93.75	57.41	32	0.81
010111100, 101001101	93.75	57.41	32	0.81
010111011, 101001010	93.75	57.41	32	0.81
010111010, 101001011	93.75	57.41	32	0.81
010011100, 101101101	89.63	61.81	32	0.81
010011010, 101101011	89.63	61.81	32	0.81
001101101, 110011100	87.78	53.75	32	0.81
001101011, 110011010	87.78	53.75	32	0.81
000111101, 111001100	81.38	44.17	32	0.81

Algorithm 1 FIT_BOOLEAN_RULES(Rules)

Require: `Rules`: dictionary mapping each node → Boolean expression (¬, ∧, ∨)
Ensure: `ValidRules`: dictionary mapping each node → set of valid candidate rules

```
Nodes ← list of all nodes in Rules
▷ 1) Compute original fixed points
A_original ← ∅
for all state s ∈ {0,1}^|Nodes| do
    if EvaluateNetwork(Rules, s) = s then
        A_original ← A_original ∪ {s}
    end if
end for
▷ 2) Main loop over each target node
for all target ∈ Nodes do
    ValidRules[target] ← ∅
    Inputs ← Nodes \ {target}
▷ 3) Generate input combinations of size 1–3
    for r = 1 to 3 do
        for all subset C ⊆ Inputs with |C| = r do
▷ 4) Generate candidate expressions for C
            Candidates ← GENERATE_CANDIDATES(C)
▷ 5) Filter by functionality on fixed points
            for all expr ∈ Candidates do
                if ∀ s ∈ A_original : EVALUATE_EXPR(expr, s) = s[target] then
▷ 6) Check attractor exactness
                    Rules' ← copy of Rules
                    Rules'[target] ← expr
                    A_new ← ∅
                    for all state t ∈ {0,1}^|Nodes| do
                        if EvaluateNetwork(Rules', t) = t then
                            A_new ← A_new ∪ {t}
                        end if
                    end for
                    if A_new = A_original then
                        ValidRules[target] ← ValidRules[target] ∪ {expr}
                    end if
                end if
            end for
        end for
    end for
end for
return ValidRules
```

Algorithm 2 GENERATE_CANDIDATES(C)

Require: C: set of r variables
Ensure: `Candidates`: set of expressions

`Candidates` $\leftarrow \emptyset$
if $|C| = 1$ **then**
    $v \leftarrow$ the single element of C
    `Candidates` $\leftarrow \{\, v, \neg v\}$
else if $|C| = 2$ **then**
    $\{x, y\} \leftarrow C$
    for all sign_x $\in \{\text{pos}, \text{neg}\}$ **do**
        for all sign_y $\in \{\text{pos}, \text{neg}\}$ **do**
            `lit_x` $\leftarrow$ (sign_x $=$ pos)?$x : \neg x$
            `lit_y` $\leftarrow$ (sign_y $=$ pos)?$y : \neg y$
            `Candidates` $\leftarrow$ `Candidates` $\cup \{$`lit_x` $\wedge$ `lit_y`, `lit_x` $\vee$ `lit_y`$\}$
        end for
    end for
else if $|C| = 3$ **then**
    $\{X, Y, Z\} \leftarrow C$
    for all each assignment of negation flags over $\{X, Y, Z\}$ **do**
        define `lit_X`, `lit_Y`, `lit_Z`
        `Candidates` $\leftarrow$ `Candidates`$\cup\{$ `lit_X`$\wedge$`lit_Y`$\wedge$`lit_Z`, `lit_X`$\vee$`lit_Y`$\vee$`lit_Z`$\}$
        `Candidates` $\leftarrow$ `Candidates` $\cup \{($`lit_X` $\wedge$ `lit_Y`$) \vee$ `lit_Z`, $($`lit_X` $\vee$ `lit_Y`$) \wedge$ `lit_Z`$\}$
    end for
end if
return `Candidates`

Algorithm 3 EvaluateNetwork(`Rules`, s)

▷Synchronous update: evaluate each rule with state s, return new state s'.

Algorithm 4 EVALUATE_EXPR(expr, s)

▷Substitute literals with values from s and evaluate → Boolean.

S11 Table. Biologically prioritized rule substitutions from the exhaustive rule-fitting output. The computational search yielded 52 dynamically admissible substitutions; the table reports the five candidates retained for detailed biological comparison, ordered by their revised biological-evidence ranking. The rank-1 BMI1 rule was selected for the fitted-network analyses. Each listed substitution preserves exactly the three desired steady states and removes the spurious synchronous limit cycle.

Original rank	Target	Candidate rule	Biological assessment
1	BMI1	(¬p53_A & ¬p53_K) | E2F1	**Selected for the fitted model.** Retains E2F1-dependent BMI1 activation, supported by direct promoter-level evidence obtained in neuroblastoma [51], and adds a p53-state-dependent derepression term. The latter is a modeling abstraction informed by the p53–miR-200c–BMI1 pathway described in mammary epithelial and breast cancer systems [53]; its NSCLC-specific implementation remains to be validated.
2	MALAT1	(¬p53_A & ¬p53_K) | Sp1	Biologically plausible alternative. Retains Sp1-dependent MALAT1 activation and adds repression by active p53 signaling [57-59].
3	miR_145	(p53_A | p53_K) & ¬MALAT1	Biologically plausible alternative. Represents either phosphorylated p53 form as active p53 and combines direct p53 induction of miR-145 with MALAT1-mediated suppression of miR-145 [54-56].
4	p53	(¬p53_A | ¬p53_K) & ¬MALAT1	Biologically plausible with a modeling caveat. Retains the original MALAT1-associated repression of p53, but expresses the phospho-state contribution as a feedback abstraction rather than a direct upstream interaction [58,59].
5	p53	(¬p53_A | ¬p53_K) & ¬KLF4	Biologically plausible with a modeling caveat. Retains experimentally supported KLF4-mediated repression of p53, while the dependence on p53 phospho-states is interpreted as feedback [42].

S12 Table. fitted rules for the 9-node network.

Node	Interactions
miR_145	p53 & !MALAT1 & !BMI1
Sp1	(BMI1) | !miR_145
MALAT1	Sp1
BMI1	(!p53_A & !p53_K) | E2F1
KLF4	!miR_145 | (E2F1 & p53)
p53	!KLF4 | !MALAT1
p53_A	!p53_K & p53
p53_K	!p53_A & p53
E2F1	MALAT1

S13 Table. Attractors results for the fitted 9-node network simulation.

Components	Steady state 1	Steady state 2	Steady state 3
miR_145	0	1	1
Sp1	1	0	0
MALAT1	1	0	0
BMI1	1	0	0
KLF4	1	0	0
p53	0	1	1
p53_A	0	0	1
p53_K	0	1	0
E2F1	1	0	0
**Attraction basin**	99.22	0.39	0.39

S14 Table. Steady state analysis across all representative asynchronous update schemes of the fitted 9-node network. Column definitions are the same as in S9 Table.

Steady state Configuration	Average Basin Attraction	SD	Count
011110001	460.87	54.98	23,107
100001010	22.47	18.46	23,107
100001100	22.47	18.46	23,107

S15 Table. Top 10 most frequent limit cycles across all representative asynchronous update schemes for the fitted 9-node network. Column definitions are the same as in S10 Table.

Limit cycle Configuration	Average Basin Attraction	SD	Count	Percent
010011100, 101101101	78.87	43.28	67	3.28
010011010, 101101011	78.87	43.28	67	3.28
000111100, 111001101	60.42	32.22	67	3.28
000111010, 111001011	60.42	32.22	67	3.28
010111010, 101001011	88.36	52.96	67	3.28
010111100, 101001101	88.36	52.96	67	3.28
000111101, 111001100	77.01	39.66	67	3.28
000111011, 111001010	77.01	39.66	67	3.28
010111011, 101001010	89.07	53.82	67	3.28
010111101, 101001100	89.07	53.82	67	3.28

S16 Table. Attractor obtained after the combined in silico perturbation of the fitted 9-node network. The perturbation fixes miR_145 and p53_A in the ON state and BMI1 in the OFF state. All 64 compatible initial configurations converge to the apoptosis attractor.

Component	Attractor 1
miR_145	1
Sp1	0
MALAT1	0
BMI1	0
KLF4	0
p53	1
p53_A	1
p53_K	0
E2F1	0
BasinSize	64
BasinPercentage	100
